# The relationship between apathy and impulsivity in large population samples

**DOI:** 10.1038/s41598-021-84364-w

**Published:** 2021-03-01

**Authors:** Pierre Petitet, Jacqueline Scholl, Bahaaeddin Attaallah, Daniel Drew, Sanjay Manohar, Masud Husain

**Affiliations:** 1 Department of Experimental Psychology, University of Oxford, Oxford, OX1 3PH UK; 2 Nuffield Department of Clinical Neurosciences, University of Oxford, Oxford, OX3 9DU UK

**Keywords:** Human behaviour, Motivation

## Abstract

Apathy and impulsivity are debilitating conditions associated with many neuropsychiatric conditions, and expressed to variable degrees in healthy people. While some theories suggest that they lie at different ends of a continuum, others suggest their possible co-existence. Surprisingly little is known, however, about their empirical association in the general population. Here, gathering data from six large studies ($n = 3755$), we investigated the relationship between measures of apathy and impulsivity in young adults. The questionnaires included commonly used self-assessment tools—Apathy Evaluation Scale, Barratt Impulsiveness Scale (BIS-11) and UPPS-P Scale—as well as a more recent addition, the Apathy Motivation Index (AMI). Remarkably, across datasets and assessment tools, global measures of apathy and impulsivity correlated positively. However, analysis of sub-scale scores revealed a more complex relationship. Although most dimensions correlated positively with one another, there were two important exceptions revealed using the AMI scale. Social apathy was mostly negatively correlated with impulsive behaviour, and emotional apathy was orthogonal to all other sub-domains. These results suggest that at a global level, apathy and impulsivity do not exist at distinct ends of a continuum. Instead, paradoxically, they most often co-exist in young adults. Processes underlying social and emotional apathy, however, appear to be different and dissociable from behavioural apathy and impulsivity.

## Introduction

The syndromes of apathy—the loss of motivation to act—and impulsivity—the tendency to act rashly, without adequate forethought—are two common conditions that are observed across a wide range of neuropsychiatric disorders^1–3^, including drug addiction^4^, ADHD^5^, schizophrenia^6^, traumatic brain injury^7^, Parkinson’s disease^8–12^, frontotemporal lobar dementia^13–15^, small vessel cereborvascular disease and vascular dementia^16,17^. It is now well established that each of these syndromes can have significant deleterious functional consequences on people who suffer from them as well as profound negative impacts on society. For example, severity of substance use is associated with both apathy and disinhibited, impulsive behaviour^4^. However, surprisingly little is understood about the relationship of each syndrome to the other^15^. Most theories that have attempted to relate apathy and impulsivity have considered them within the context of models of frontal lobe function or dopaminergic system but have come to distinctly different conclusions.

Traditionally, frontal lobe function has been conceptualised as resting on separate domains (e.g., executive functions, personality/social behaviour, motivation, etc.), each of which might be underpinned by different parts of frontal cortex (e.g., dorso-lateral prefrontal cortex, orbitofrontal cortex, anterior cingulate cortex) and their connections to different regions within the basal ganglia^18–21^. One important consequence of such a “circuit-specific” framework is that frontal lobe *dysfunction* can also theoretically be fractionated along the same behavioural axes^22^. For example, impulsivity and apathy have classically been associated with orbitofrontal cortex (OFC) and anterior cingulate cortex (ACC) dysfunction respectively^2,23^. A second important consequence is that neuropsychiatric disorders should mostly occur independently from one another when lesions are focal, and co-exist only when damage is more extensive, involving more than one circuit or system. Thus, despite their possible co-occurrence, it should be possible to identify brain regions uniquely associated with any given neuropsychiatric syndrome. This experimental approach has indeed proven successful in frontotemporal dementia (FTD) where independent, unique, anatomical substrates of apathy and impulsivity have been isolated^13,24,25^.

However, within the same clinical population (FTD), other studies have questioned this “circuit-specific” view, reporting common brain regions associated with both apathy and impulsivity^14,15,26^. Is it possible that, in addition to being partly dissociable, these two neuropsychiatric disorders also share some common neural substrates? The “dopamine overdose hypothesis” within the Parkinson’s disease (PD), schizophrenia, and addiction literature is indeed compatible with this view. According to this model, apathy and impulsivity may originate from opposing levels of dopaminergic dysregulation within the same neural networks involved in motivated behaviour^10,11,27,28^. Specifically, impulsivity might be associated with too much dopamine, whereas apathy might be due to too little. Support for this framework in PD comes from both cross-sectional and longitudinal studies of patients^8,9,29–32^.

For example, some patients with PD can develop pathological impulse control disorders (ICDs)—characterised by, for example, gambling, hypersexuality and other impulsive behaviours—when their dose of dopaminergic medication is increased, but this can resolve when the dose is subsequently decreased^30,31^. Conversely, sudden reduction in dopaminergic drug dosage following successful implantation of deep brain stimulation electrodes for treatment of PD, can lead to severe apathy, but this can be reversed by the introduction of a dopaminergic agonist^29^. The fact that patients can behave either impulsively or apathetically as a function of their dopamine agonist dosage provides evidence that the two conditions exist at opposite extremes of a continuous spectrum mediated by tonic cortico-striatal dopamine^10,33^.

The two (non-exclusive) candidate mechanisms discussed above make distinct predictions in terms of the possible co-occurrence of apathy and impulsivity in neurological patients. If the two disorders originate from the disruption of parallel circuits that may or may not partially overlap (e.g., “circuit-specific” model), then their incidence should be either independent from one another, or positively correlated. If, however, they implicate opposite neuromodulatory dysregulations of the same networks (e.g., “dopamine overdose hypothesis”), then apathy and impulsivity should never be found concurrently, and should negatively correlate (within- and across- subjects). Thus, the study of the relationship between the manifestations of these two neuropsychiatric disorders can indirectly inform investigations into the potential segregation of their neurobiological basis.

Large scale cross-sectional studies performed in neurological and psychiatric populations have predominantly supported the “circuit-specific” account. Apathy and impulsivity have mostly been described as separate, but nevertheless positively correlated, factors^5,14,15,34^, even in PD patients^35^. Although these observations seemingly provide little support for these constructs being at different ends of a single axis (as formulated by the “dopamine overdose hypothesis”), the following potential confound is worth considering.

Apathy and impulsivity are umbrella clinical terms that tend to encapsulate various aspects of behaviour, which makes them multidimensional in nature^1,10,36,37^. For example, questionnaire measures of apathy have isolated at least four dissociable subdomains in which loss of motivation can be expressed: goal-directed behaviour, cognitive activities, social interactions, and emotional responses^36,38,39^. Similarly, self-report measures of impulsivity^40,41^ usually distinguish between different domains: short deliberation style, lack of premeditation/planning, lack of perseverance and positive/negative urgency (i.e., tendency to act rashly under extreme positive/negative emotions), attentional impulsiveness, etc. The various constituent phenomenological manifestations of these syndromes might therefore involve separable, distinct mechanisms, some of which may be organised along parallel circuits, and others along an apathy-impulsivity axis. Thus, the fine-grained dissection of the components of apathy and impulsivity, and the characterisation of their relationship with one another, are essential prerequisites to any investigation of their brain bases and therapeutic treatments.

To our knowledge, the precise relationships between sub-components of apathy and impulsivity, as assessed by self-report questionnaire measures, remain unclear. Here, we use large questionnaire datasets collected in the general population to investigate this question. Consistent with findings in neuropsychiatric populations, we show that overall the total scores of the apathy and impulsivity questionnaires systematically correlate positively. However, deconstructing these scores into their constituent sub-domains revealed the presence of overlapping, orthogonal, and even anti-correlated dimensions. More specifically, two apathy dimensions—as assessed by the recently developed Apathy Motivation Index^36^ (AMI)—clearly stood out in the analyses: emotional apathy and social apathy. Emotional apathy was orthogonal to any other sub-domain, potentially suggesting a completely dissociable anatomical substrate; whereas social apathy was the only apathy dimension to negatively correlate with some impulsive manifestations. Taken together, these findings provide support for both types of candidate mechanism, but suggest that they may be implicated in different manifestations of apathy and impulsivity.

## Results

### Self-report measures of apathy and impulsivity largely overlap

**Figure 1:**
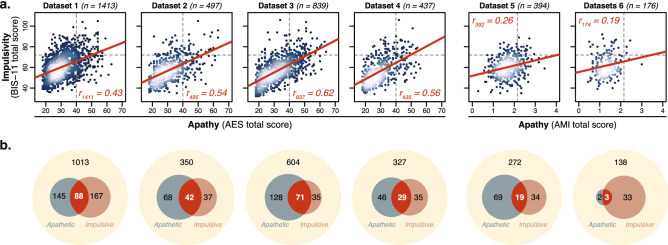
Apathy (AES, AMI) and impulsivity (BIS-11) are positively correlated. (**a**) In all four open datasets^42–45^, a positive correlation was observed between the total score of the AES and BIS-11. In the two datasets including the AMI instead of the AES (Datasets 5–6), a similar, although weaker, relationship was observed. Lighter colours represented higher density. (**b**) Venn diagrams highlighted a large proportion of people who qualified as both apathetic and impulsive according to standard cut-offs (dotted grey lines in panel a; AES total: 40^46^; BIS-11 total: 72^47^; AMI total: 2.15^36^).

First, the relationship between apathy and impulsivity was investigated in four large open datasets which all used the AES and BIS-11. Significant positive correlations were observed between the total score of these two questionnaires in all of these studies (Dataset 1: $r_{(1411)}=0.43$, Dataset 2: $r_{(495)}=0.54$, Dataset 3: $r_{(836)}=0.62$, Dataset 4: $r_{(435)}=0.56$, all $p<10^{-15}$; $r_{(3183)}=0.51$ when pooling Datasets 1-4 together; Fig. 1a). On average across the four datasets, $29.2\%$ ($s.d.: 8.2\%$) of the variance of a questionnaire could be explained by the other. Using standard clinical cut-offs to categorise individuals according to their apathy and impulsivity status highlighted a large degree of overlap between the two constructs (Fig. 1b). According to these cut-offs, one in 14 individuals ($7.4\%$, $s.d.: 1.2\%$) qualified as both apathetic *and* impulsive. On average across the four datasets, more than a third of apathetic individuals were also categorised as impulsive ($37.6\%$, $s.d.: 1.3\%$), and half of impulsive individuals were also categorised as apathetic ($50.0\%$, $s.d.: 13.7\%$; Fig. 1b).

### All AES and BIS-11 sub-scales positively correlate

**Figure 2:**
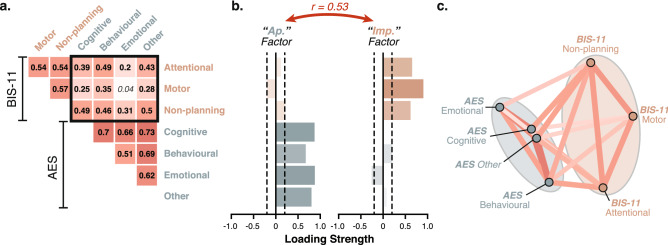
All dimensions of the AES (apathy) and BIS-11 (impulsivity) positively correlate. (**a**) The correlation matrix of the sub-scale scores of the AES and BIS-11 questionnaires showed positive correlations only ($n=3185$; Datasets 1–4). Significant pairwise correlations are shown in bold (Bonferroni-corrected). The black square highlights cross-questionnaire associations. (**b**) Factor decomposition (EFA) of this matrix uncovered dissociable, nevertheless positively correlated ($r=0.53$), “Apathy” and “Impulsivity” factors. (**c**) In this network plot, sub-scale scores that are more highly correlated (positively or negatively) appear closer together and are joined by stronger paths (non-significant paths removed). Paths are also coloured by their sign (red for positive and blue negative). Ellipses are drawn around the sub-scores included in a factor. Note, here all paths are positive, even those connecting the two factors together. See also Fig. S1 for the same plots split by dataset.

In order to investigate the factors driving the association between AES and BIS-11, we calculated the pairwise correlation matrix between sub-scale scores (pooling all four datasets together, $n=3185$; see Fig. S1 for similar results in individual datasets). We reasoned that, given the known multidimensional nature of both psychopathological constructs (AES: emotional, behavioural, cognitive, other; BIS-11: attentional, motor, non-planning impulsiveness), some of the underlying factors may overlap (positive correlation), while others may actually be organised along an apathy-impulsivity axis (negative correlation). Although there was strong evidence for the former, the latter was unsupported by the data. All sub-scale scores of the AES and BIS-11 clustered together (positive correlations), and we did not observe any evidence of an apathy-impulsivity axis (no negative correlation; Figs. 2 and S1).

Factor decomposition (EFA) provided an objective, formal description of the sub-score correlation matrix. The best solution retained two distinct factors ($\chi ^2_{(8)}=179.14$, $p<10^{-33}$) that mapped onto the two constructs assessed by the respective questionnaires: apathy and impulsivity (Fig. 2). Consistent with the total score results (Fig. 1), however, these two factors were positively correlated with one another ($r=0.53$).

**Figure 3:**
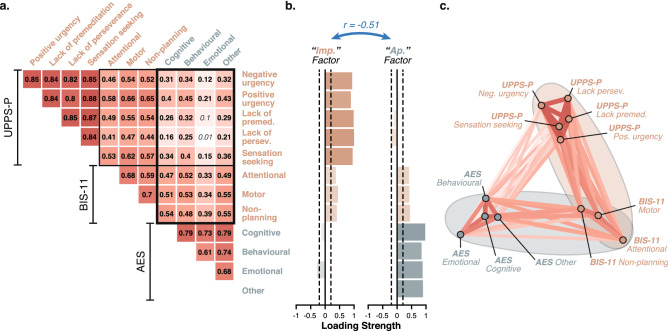
BIS-11 sub-scales contribute equally to apathy and impulsivity. (**a**) The correlation matrix of the sub-scale scores of the UPPS-P, BIS-11 and AES questionnaires showed positive correlations only (Dataset 3, $n=809$ complete observations). Significant pairwise correlations are shown in bold (Bonferroni-corrected). The black square highlights cross-questionnaire associations. (**b**) Factor decomposition (EFA) of this matrix uncovered dissociable, anti-correlated ($r=-0.51$), “Apathy” and “Impulsivity” factors. BIS-11 sub-scales contributed equally to both factors. (**c**) The network plot showed a characteristic isosceles clustering. More highly correlated (positively or negatively) appear closer together and are joined by stronger paths (non-significant paths removed). Paths are also coloured by their sign (red for positive and blue negative). Ellipses are drawn around the sub-scores included in a factor.

### Analysis of UPPS-P with BIS-11 and AES data

Next, we asked whether adding another impulsivity questionnaire to the analysis would improve our ability to dissociate apathy from impulsivity. In addition to the AES and BIS-11, Dataset 3^43^ included the UPPS-P which is a more extensive measure of impulsivity. All three questionnaires were positively correlated (total scores; all $r_{(836)}>0.35$, $p<10^{-24}$) but they differed in the strength of their association with one another. The AES was more strongly associated with the BIS-11 than the UPPS-P ($z = 11.02$, $p<10^{-4}$), the UPPSP-P was more strongly associated with the BIS-11 than the AES ($z=-12.32$, $p<10^{-4}$), and the BIS-11 was equally associated with the other two questionnaires ($z = -1.52, \ p = 0.13$; see also SI for partial correlations). This is illustrated as an isosceles triangular clustering in Fig. 3c (note how the BIS-11 is roughly equidistant to the UPPS-P and AES, while the AES:UPPS-P segments are longer). This structure is revelatory: it suggests that the psychopathological construct assessed by the BIS-11 is conceptually as close to an apathy questionnaire (AES) as it is to the same construct measured by a different instrument (UPPS-P).

Factor decomposition of the sub-scale correlation matrix for Dataset 3 offered an objective formalisation of this structure. Two factors ($\chi ^2_{(43)}=429.6$, $p<10^{-64}$), predominantly dominated by UPPS-P and AES sub-scales, mapped onto impulsivity and apathy respectively (Fig. 3b). The BIS-11 sub-scales, however, contributed positively and equally to both factors, highlighting its conceptual overlap with both psychopathological constructs. Intriguingly, when the UPPS-P was incorporated into the correlation matrix, the “Apathy” and “Impulsivity” factors became anti-correlated ($r=-0.51$), mainly because emotional apathy, as assessed by the AES (two items), contributed in opposite directions to the two constructs. We interpreted this as evidence that part of the construct captured by the BIS-11 was organised along an apathy-impulsivity axis but that this structure could not be revealed with the AES/BIS-11 combination alone.

### A clear apathy-impulsivity axis uncovered in the social domain

In Dataset 5, the Apathy Motivation Index^36^ (AMI)—a different self-report measure of apathy developed in our laboratory and optimised for healthy people—was collected alongside the BIS-11 as part of a large online study in healthy people ($n=394$; collected with Prolific). Once again, the total scores of the two questionnaires correlated positively (AMI vs. BIS-11; $r_{(392)}=0.26$, $p<10^{-6}$; Fig. 1). This association, however, was significantly weaker than previously observed between the AES and BIS-11 total scores ($z=-5.56$, $p<10^{-4}$).

**Figure 4:**
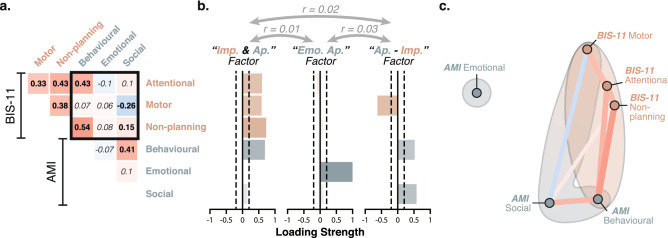
An apathy-impulsivity axis can be uncovered with the AMI and BIS-11. (**a**) The correlation matrix of the sub-scale scores of the AMI and BIS-11 questionnaires showed positive and negative associations (Dataset 5, $n=394$). Significant correlations are shown in bold (Bonferroni-corrected significance threshold: $p<0.003$). The black square highlights cross-questionnaire associations. (**b**) Factor decomposition (EFA) of this matrix yielded 3 un-correlated factors (Apathy-Impulsivity overlap, Emotional apathy, Apathy-Impulsivity axis). (**c**) The network plot is shown for visualisation purposes. More highly correlated (positively or negatively) appear closer together and are joined by stronger paths (non-significant paths removed). Paths are also coloured by their sign (red for positive and blue negative). Ellipses are drawn around the sub-scores included in a factor. See also Fig. S3 for replication in an independent dataset.

The weaker association was explained by the presence of a negative apathy-impulsivity axis within the cross-questionnaire sub-scale correlation matrix (Fig. 4). More specifically, social apathy—a dimension that is absent from the AES questionnaire—negatively correlated with motor impulsiveness (Fig. 4). Thus, within these domains, people reported behaving either apathetically or impulsively, but crucially not both. In addition, it is worth highlighting that (unlike in the AES), the emotional apathy sub-scale of the AMI (six questions out of 18; e.g., “I feel awful if I say something insensitive.”) was not significantly associated with any other dimension, demonstrating the specificity of the construct captured by this sub-scale.

Factor decomposition (EFA) uncovered three dissociable constructs (Fig. 4) that confirmed this interpretation: (1) An “Apathy-Impulsivity overlap” factor containing all sub-scales of the BIS-11 as well as the behavioural sub-scale of the AMI; (2) An “Emotional Apathy” factor (emotional subscale of the AMI); (3) An “Apathy-Impulsivity axis” factor made of the behavioural and social sub-scales of the AMI positively, and the motor sub-scale of the BIS-11 negatively. This structure was further replicated in Dataset 6 ($n=176$; Figs. 1 and S3), and was also observed when performing the EFA at the item level (Fig. S4).

## Discussion

Apathy and impulsivity are common multi-dimensional syndromes of motivation that are associated with many different disorders^1–3^. Competing models make distinct predictions regarding their possible association. The “circuit-specific” hypothesis^22^ proposes that the two syndromes are independent of one another and predicts no or possibly positive associations (depending on the extent of circuits involved by the pathology). By contrast, the “dopamine overdose” hypothesis^27^ places these constructs at opposite ends of a single continuum, thereby predicting a negative correlation between apathy and impulsivity. Here, using data collected in large population samples, we investigated the relationship between the two syndromes and their constituent dimensions.

Across the four largest datasets^42–45^ (total $n=3185$), the most commonly used measures of apathy (AES^38^) and impulsivity (BIS-11^40^) were systematically positively correlated. This was the case both when quantifying these traits as single monolithic entities (total scores; Fig. 1), and when parcellating them into their proposed constituent dimensions (AES: cognitive/behavioural/emotional/other; BIS-11: motor/non-planning/attentional impulsivity; Fig. 2). The result is consistent with recent observations in clinical populations such as ADHD^5^, frontotemporal lobar dementia^14,15^, and PD^35^. The conceptual overlap between AES and BIS-11 was further demonstrated by using the UPPS-P^41^ as an additional, more extensive, measure of impulsivity (Dataset 3). In this case, apathy and impulsivity still loaded onto distinct, this time negatively correlated, factors (dominated by the AES and UPPS-P respectively) but importantly the BIS-11 contributed equally to both (Fig. 3). This suggests that part of the construct captured by the BIS-11 was organised along an apathy-impulsivity axis that could not be revealed with the AES alone.

Is the magnitude and direction of the association between apathy and impulsivity, at least partly, determined by the assessment instrument used? To answer this question, we analysed a fifth dataset including an alternative measure of apathy—the Apathy Motivation Index^36^—in addition to the BIS-11. Once again, the total scores of the two questionnaires correlated positively. However, factor decomposition revealed three distinct trait modes in which overlap, orthogonality, and anti-correlation were expressed between sub-components of apathy and impulsivity (Figs. 4, S3, S4). The first one captured commonalities between a lack of self-initiated goal-directed behaviour (behavioural apathy) and all sub-components of impulsivity. People who scored high on this dimension reported having difficulties self-initiating and sustaining effortful activities, which was accompanied by a tendency to think and act rashly, perhaps as a result of poor planning abilities. The second factor was entirely dissociable from all other apathy and impulsivity manifestations and captured emotional blunting. Finally, the third factor corresponded to an apathy-impulsivity axis with diminished behavioural and social activities on one end, and spontaneous “happy-go-lucky” response style on the other. Thus, within this mostly social domain, individuals reported being either withdrawn (apathetic) or disinhibited (impulsive), but crucially not both. Importantly, these three factors showed rather weak associations with one another (Figs. 4, S3, S4), indicating that they corresponded to segregated clusters of individuals.

Emerging data suggest that neither apathy, nor impulsivity are monolithic entities^1,2^. Our analyses demonstrated that, in fact, they show distinct relationships to one another depending crucially on the domain considered (e.g., goal-directed behaviour, emotional sensitivity, social behaviour). As a result, the questionnaires used and the specific domains they encompass have a large influence on the direction and magnitude of the association that can be expected between apathy and impulsivity. For example, because it does not include a social sub-scale, the AES alone was not able to uncover negative associations with impulsivity (Fig. 1). Similarly, because the emotional sub-scale of the AES (2 items out of 18) is under-represented in the total score, the likelihood of observing a positive association between apathy and impulsivity was inflated with the AES.

What are the implications of the three-factor structure identified with the AMI/BIS-11 combination in terms of possible brain basis and therapeutic intervention? Disruption of goal–directed decision-making is now recognised to be an important aspect of neuropsychiatric disorders^42,43,48–50^. Because it has typically been described in experimental contexts in which individuals are forced to act/decide (e.g., two-step reinforcement learning task, devaluation task), the counterpart to this disruption is often thought to be an increase in habitual responding. This explains why it has predominantly been linked to addiction or compulsivity where the balance between these two modes of responding is likely to play a key role^51^. This “goal-directed versus habitual” framework, however, is constrained by the type of laboratory manipulation that gave rise to it^52^. Our analyses of population data suggest that, in a naturalistic setting, a diminution of self-generated actions (behavioural apathy) and an increase in automatic responding to salient external stimuli (impulsivity) may constitute *alternative* responses to deficits of goal-directed control. Simply put, these individuals may have difficulties generating/selecting behavioural options for themselves, but may nevertheless be motivated to act when simple courses of actions are readily available or more complex ones are planned or proposed by others. Thus, we hypothesize that a disruption of common brain networks involved in self-generated, goal-directed control^53^ may, at least in part, account for the positive association between both behavioural apathy and impulsivity.

Perhaps one reason why apathy and impulsivity have traditionally been considered to be opposite to one another^10,11,27^ is because they appear to antagonise each other within the social domain, i.e., the dimension that is easiest to assess from an external perspective, whether it is from the view point of the care giver or a clinician. Individuals who reported having difficulties initiating actions both for themselves (e.g., “When I decide to do something, I am *not* able to make an effort easily.”; behavioural apathy) and for others (e.g., “I *do not* suggest activities for me and my friends to do.”; social apathy) were the least impulsive on the BIS-11 motor sub-scale (e.g., “I am happy-go-lucky.”). Thus, it is the generalisation of the motivational disorder beyond self-generated actions and into the social domain that clearly distinguished this aspect of apathy from the component of apathy that overlapped with impulsivity (“Apathy-Impulsivity overlap” factor).

This differentiation of these two aspects of apathy might be underpinned by distinct primary deficits: one being disrupted goal-directed control and the other related to altered subjective valuation. A wealth of evidence now supports the existence of a single valuation system in the brain (predominantly relying on ventromedial prefrontal cortex and ventral striatum) that uses a “common neural currency” to assign a subjective value to stimuli of any nature^54–56^, social or not^57,58^. Tonic dopamine is known to have an important modulatory influence on this system. It is possible that opposing dysregulation of this dopaminergic modulation may be the underlying mechanism responsible for the apathy-impulsivity axis that spans the behavioural and social domains. Consistent with the “dopamine overdose hypothesis”^10,11,27^, too much or too little ventral striatal dopamine may promote over- or under- sensitivity to reward of any kind (social or not), thereby pushing people to behave either apathetically or impulsively. Note that, according to this proposed mechanism, individuals might be fundamentally unwilling/overwilling to act for both self and others because of an under-/over- estimation of potential benefits respectively. This feature distinguishes it from the previous proposed mechanism in which deficits of goal-directed control might be rescued by others in a social context.

Finally, our analyses identified diminished emotional sensitivity, as assessed by the AMI emotional sub-scale (6 items out of 18; e.g., “I *do not* feel awful if I say something insensitive.”), as mostly dissociable from other apathy and impulsivity manifestations, suggesting a potentially distinct organic basis. This finding is consistent with existing accounts describing the emotional system as a self-contained network capable of influencing other functions (e.g., motivation, attention) by interacting with the brain networks dedicated to these functions^59,60^. The specific disruption of the emotion system may therefore constitute an indirect entry into apathy that has more in common with disorders of affective empathy^61,62^ than with the other two mechanisms proposed above^1,3,63^ (disruptions of goal-directed control and subjective valuation). Consistent with this view, cases of isolated emotional apathy have been linked to specific emotional recognition deficits in amyotrophic lateral sclerosis (ALS) patients^64^.

The results presented here also have important methodological implications for future studies of apathy and impulsivity. A large body of the literature has used cross-sectional correlations between single questionnaire total scores (e.g. AES, AMI, BIS-11, UPPS-P), and behavioural (e.g. performance on a task, computational parameter, etc.) or physiological (e.g. dopamine signaling, functional connectivity, white matter integrity, etc.) measures of interest to characterise putative psychological or neurobiological mechanisms underlying apathy or impulsivity^65–70^. Similarly, other studies have used a categorical approach in which two groups, defined based on total score cut-offs, are compared to one another in regards to some other variable of interest^71,72^. From a methodological perspective, the associations reported in this paper suggest that it might be important to control for apathy when investigating impulsivity, and vice versa (e.g., using partial correlation methods). In the absence of such corrections, one may risk attributing a certain mechanism to one construct when in fact it is relevant to the other. Further, the results presented here highlight the importance of parcellating these disorders into their possible manifestations, as different domains of apathy (behavioural and social, emotional) show distinctly different relationships to impulsivity, suggesting the involvement of dissociable underlying brain mechanisms.

In conclusion, our analyses of several large datasets support the existence of cross-diagnostic modes of covariation that may be underpinned by distinct brain mechanisms (deficits of goal-directed control, under-/over- sensitivity to reward, altered emotional/empathic processing). This challenges existing diagnostic boundaries between apathy and impulsivity, and suggests that future studies should investigate these constructs in association, dissociation, or isolation, depending on the domain considered.

## Methods

### Data collection and participants

#### Open datasets

Data available in four large-scale online studies were examined^42–45^ ($n=3185$ in total, 18-76 years of age; Table 1) . All participants, recruited via *Amazon Mechanical Turk* (AMT; https://www.mturk.com), gave electronic informed consent and were compensated for their time. The studies were approved by their respective local ethics committee (Dataset 1: New York University Committee on Activates Involving Human Subjects; Dataset 2: University College London Research Ethics Committee; Dataset 3: Trinity College Dublin School of Psychology Research Ethics Committee; Dataset 4: Harvard Committee on the Use of Human Subjects), and carried out in accordance with the relevant guidelines and regulations.

Participants performed the experiment from home. Details of exclusion criteria can be found in the respective publications^42–45^. These criteria were defined a priori to ensure data quality, and included: (1) task-related criteria (e.g. performance threshold), (2) questionnaire-related criteria (e.g. random catch items to ensure participants were paying attention), and (3) IQ-related criteria (e.g., failure to solve any of the Raven’s Standard Progressive Matrices^73^). Beyond the self-report questionnaire measures, no information about psychiatric diagnosis or medication was recorded in any of the four studies. As a result, the samples considered here may include participants suffering from diagnosed or un-diagnosed psychiatric or neurological condition, who may or may not have been treated with neuroactive medication at the time. This might be a weakness but the heterogeneity is also a potential strength as it ensured large inter-individual variability, a prerequisite to study co-variation between self-report measures of apathy and impulsivity.

**Table 1:** Demographics characteristics.

Dataset	F/M	Age	AES	AMI	BIS-11	**UPPS-P**
1. Gillan et al. ^42^	823/590	33.0 (10.8)	31.5 (8.2)	–	61.1 (11.2)	–
2. Rouault et al. ^44^	257/240	35.6 (10.6)	32.3 (9.7)	–	58.3 (12.2)	–
3. Patzelt et al. ^43^	377/461	36.1 (10.3)	32.5 (9.9)	–	57.1 (12.4)	111 (26)
4. Seow and Gillan ^45^	228/209	35.3 (10.3)	30.4 (8.8)	–	56.9 (12.6)	–
5. Online dataset	195/194 (5)	28.0 (6.0)	–	1.72 (0.52)	60.4 (9.8)	–
6. Laboratory dataset	89/87	24.8 (4.3)	–	1.33 (0.41)	61.9 (10.8)	–

The table shows the number of females (F) and males (M) included in each study (“Prefer not to say” in parenthesis). Group mean total scores (standard deviation in parenthesis) are shown for each questionnaire included in a dataset.

#### Additional datasets

Two other datasets from our laboratory were used in this paper (Table 1). They all included self-report questionnaire measures in addition to behavioural task data. The larger one (Dataset 5; $n=394$) was collected online using *Prolific* (https://www.prolific.co), while the smaller one (Dataset 6; $n=176$) was collected in person in the laboratory. Both studies were approved by the University of Oxford ethics committee, and carried out in accordance with the relevant guidelines and regulations. All participants gave informed consent (electronically for Datasets 5; in person for Dataset 6) prior to the experiment.

Inclusion criteria of the online dataset were comparable to those of the four open datasets. The laboratory sample was representative of the population studied by most cognitive psychology experiments: un-medicated students or young professionals, who reported no history of psychological or neurological condition. Of interest, it was noted that participants who came to the laboratory (Dataset 6) tended to have lower total apathy scores (AMI) than participants who participated online (Dataset 5; $t_{(414)}=9.55$, $p<10^{-15}$; see *SI*; Fig. S2).

### Self-report questionnaire measures

The open datasets used in this paper included at least nine self-report questionnaires spanning various psychiatric symptoms (e.g. alcohol addiction, eating disorders, obsessive-compulsive disorder, depression, anxiety, schizotypy, social anxiety, impulsivity, apathy) in order to extract trans-diagnostic dimensions, and investigate their associations with neurocognitive performance^42–45^. Similarly, the additional online dataset (Dataset 5) included 11 self-report questionnaire measures probing alcohol addiction, obsessive-compulsive disorder, depression, anxiety, schizotypy, social anxiety, alexithymia, fatigue, mindfulness, impulsivity, and apathy.

The central objective of this work was to assess the degree of association/dissociation between commonly recognised constituent dimensions of apathy and impulsivity. Thus, we focused on the following stand-alone self-report questionnaires:

- *Apathy Evaluation Scale*^38^ (AES) 18-item questionnaire scored on a 4-point Likert scale (total scores ranging from 18 to 72), subdivided into 4 sub-scales (emotional apathy, behavioural apathy, cognitive apahty, other);
- *Apathy Motivation Index*^36^ (AMI) 18-item questionnaire scored on a 5-point Likert scale (total scores ranging from 0 to 4), sub-divided into 3 sub-scales (emotional apathy, behavioural apathy, social apathy);
- *Barratt Impulsiveness Scale*^40^ (BIS-11) 30-item questionnaire, scored on a 4-point Likert scale (total scores ranging from 30 to 120), sub-divided into 3 sub-scales (attentional impulsiveness, motor impulsiveness, non-planning impulsiveness);
- *Urgency, Premeditation (lack of), Perseverance (lack of), Sensation Seeking, Positive Urgency, Impulsive Behavior Scale*^41^ (UPPS-P) 59-item questionnaire scored on a 4-point Likert scale (total scores ranging from 59 to 236), sub-divided into the 5 sub-scales (negative urgency, lack of premeditation, lack of perseverance, sensation seeking, positive urgency).

A complete item-by-item description of the questionnaires and their scoring is provided in SI. For both total and sub-scale scores, larger values indicate more severe apathy or impulsivity. When fractionating total scores into their constituent sub-scores, the original factor structure of individual questionnaires were used. This approach ensured that the dimensions considered in this paper matched those used by most studies using these self-report measures. Further, a Confirmatory Factor Analysis (CFA) was performed for each questionnaire to check the validity of the original factor structure (see SI). The relationship between the AES and AMI questionnaires was not investigated in this paper because the datasets considered included one or the other but not both. However, this relationship was investigated elsewhere^36^.

### Data analysis

Data handling and analysis were performed in the free software R^74^. All *p*-values are two-sided unless specified otherwise. The Venn diagrams in Fig. 1 were generated with a cut-off of 40 for the AES (average of the low and high cut-offs^46^), 72 for the BIS-11^47^, and 2.15 for the AMI (average of the high and low cut-offs^36^). All correlations were quantified using bivariate Pearson correlation coefficient, and analysed statistically with t-tests (p-statistic threshold adjusted for multiple comparison using Bonferroni correction). The percentage of variance of a dependent variable explained by an independent variable was subsequently quantified using R-squared. Correlation coefficients were compared using Hittner’s modification of Fisher’s r-to-z procedure^75^, implemented in the package *cocor* (http://comparingcorrelations.org).

To provide a better visualisation of correlation matrices, network plots were generated with the function “network_plot” of the package *corrr* (https://github.com/tidymodels/corrr). This approach uses multidimensional clustering (multidimensional scaling of the absolute values of the correlations) to determine the proximity of variables. It offers a simple and intuitive way to visualise clusters in the data, i.e., groups of variables that more strongly co-vary together (positively or negatively).

In order to investigate the possibility that a more parsimonious latent trans-questionnaire structure could explain sub-scale scores and their relationships with one another, Exploratory Factor Analyses (EFA) were conducted using a maximum likelihood estimation (MLE) procedure. These analyses were performed using the *factanal* function from the *Psych* package in R (https://personality-project.org/r/psych). An oblique rotation (Promax) was used to allow for factors to be correlated. Parallel analysis (implemented in the *nFactors* package in R) was used to determine the number of factors to keep^76^. This method contrasts the data eigenvalues to those of random data of the same size generated from a Monte-Carlo simulations, and retains factors for which this difference is positive. To confirm that the number of factors selected was sufficient, a $\chi ^2$-test was performed for each EFA. For visualisation purposes, factors were manually drawn on top of the network plots in Figs. 2, 3 and 4. EFAs were only performed if the ratio of variable-to-participants was at least 1 : 10. Meeting this statistical criterion required pooling Datasets 6 and 7 together when the EFA was performed at the item level for the AMI and BIS-11 questionnaires (i.e., 48 variables; Fig. S4).

## Supplementary Information

Supplementary Information.

Supplementary Information.

Supplementary Information.

Supplementary Information.

Supplementary Information.

Supplementary Information.

Supplementary Information.

## Data Availability

All data generated or analysed during this study are available from the respective authors^42–45^ (Datasets 1–4), or from the corresponding author (Datasets 5-6).

## References

[1] Dalley JW, Robbins TW (2017). Fractionating impulsivity: neuropsychiatric implications. Nat. Rev. Neurosci..

[2] Husain M, Roiser JP (2018). Neuroscience of apathy and anhedonia: a transdiagnostic approach. Nat. Rev. Neurosci..

[3] Starkstein SE, Brockman S (2018). The neuroimaging basis of apathy: empirical findings and conceptual challenges. Neuropsychologia.

[4] Verdejo-Garcìa A, Rivas-Pérez C, López-Torrecillas F, Pérez-Garcìa M (2006). Differential impact of severity of drug use on frontal behavioral symptoms. Addict. Behav..

[5] Torrente F (2011). Not always hyperactive?: elevated apathy scores in adolescents and adults with adhd. J. Atten. Disord..

[6] Bortolon C, Macgregor A, Capdevielle D, Raffard S (2018). Apathy in schizophrenia: a review of neuropsychological and neuroanatomical studies. Neuropsychologia.

[7] Worthington A, Wood RL (2018). Apathy following traumatic brain injury: a review. Neuropsychologia.

[8] Ahearn DJ, McDonald K, Barraclough M, Leroi I (2012). An exploration of apathy and impulsivity in Parkinson disease. Curr. Gerontol. Geriatr. Res..

[9] Leroi I (2012). Apathy and impulse control disorders in Parkinson’s disease: a direct comparison. Park. Relat. Disord.

[10] Sinha N, Manohar S, Husain M (2013). Impulsivity and apathy in Parkinson’s disease. J. Neuropsychol..

[11] Sierra M (2015). Apathy and impulse control disorders: Yin and Yang of dopamine dependent behaviors. J. Park. Dis..

[12] Houeto JL, Magnard R, Dalley JW, Belin D, Carnicella S (2016). Trait impulsivity and anhedonia: two gateways for the development of impulse control disorders in Parkinson’s disease?. Front. Psychiatry.

[13] Zamboni G, Huey ED, Krueger F, Nichelli PF, Grafman J (2008). Apathy and disinhibition in frontotemporal dementia. Neurology.

[14] Lansdall CJ (2017). Apathy and impulsivity in frontotemporal lobar degeneration syndromes. Brain.

[15] Passamonti L, Lansdall CJ, Rowe JB (2018). The neuroanatomical and neurochemical basis of apathy and impulsivity in frontotemporal lobar degeneration. Curr. Opin. Behav. Sci..

[16] Reyes S (2009). Apathy. Neurol..

[17] Lohner V, Brookes RL, Hollocks MJ, Morris RG, Markus HS (2017). Apathy, but not depression, is associated with executive dysfunction in cerebral small vessel disease. PLoS ONE.

[18] Alexander GE, DeLong MR, Strick PL (1986). Parallel organization of functionally segregated circuits linking basal ganglia and cortex. Annu. Rev. Neurosci..

[19] Middleton FA, Strick PL (2002). Basal-ganglia ‘projections’ to the prefrontal cortex of the primate. Cereb. Cortex.

[20] Stuss DT, Alexander MP (2007). Is there a dysexecutive syndrome? Philos. Trans. R. Soc. B Biol. Sci..

[21] DeLong MR, Wichmann T (2007). Circuits and circuit disorders of the basal ganglia. Arch. Neurol..

[22] Mega MS, Cummings JL (1994). Frontal-subcortical circuits and neuropsychiatric disorders. The J. Neuropsychiatry Clin. Neurosci..

[23] Jonker FA, Jonker C, Scheltens P, Scherder EJ (2015). The role of the orbitofrontal cortex in cognition and behavior. Rev. Neurosci..

[24] Powers JP (2014). White matter disease contributes to apathy and disinhibition in behavioral variant frontotemporal dementia. Cogn. Behav. Neurol. Off. J. Soc. for Behav. Cogn. Neurol..

[25] Sheelakumari R (2019). Neuroanatomical correlates of apathy and disinhibition in behavioural variant frontotemporal dementia. Brain Imag. Behav..

[26] Rosen HJ (2005). Neuroanatomical correlates of behavioural disorders in dementia. Brain.

[27] Vaillancourt DE, Schonfeld D, Kwak Y, Bohnen NI, Seidler R (2013). Dopamine overdose hypothesis: evidence and clinical implications. Mov. Disord..

[28] Kirschner M, Rabinowitz A, Singer N, Dagher A (2020). From apathy to addiction: insights from neurology and psychiatry. Prog. Neuro-Psychopharmacology Biol. Psychiatry.

[29] Thobois S (2010). Non-motor dopamine withdrawal syndrome after surgery for Parkinson’s disease: predictors and underlying mesolimbic denervation. Brain.

[30] Rabinak CA, Nirenberg MJ (2010). Dopamine agonist withdrawal syndrome in Parkinson disease. Arch. Neurol..

[31] Pondal M (2013). Clinical features of dopamine agonist withdrawal syndrome in a movement disorders clinic. J. Neurol. Neurosurg. Psychiatry.

[32] Weintraub D (2010). Impulse control disorders in parkinson disease: a cross-sectional study of 3090 patients. Arch. Neurol..

[33] Volkmann J, Daniels C, Witt K (2010). Neuropsychiatric effects of subthalamic neurostimulation in parkinson disease. Nat. Rev. Neurol..

[34] Carvalho JO, Ready RE, Malloy P, Grace J (2013). Confirmatory factor analysis of the frontal systems behavior scale (frsbe). Assessment.

[35] Baig F (2019). Impulse control disorders in parkinson disease and rbd. Neurology.

[36] Ang YS, Lockwood P, Apps MA, Muhammed K, Husain M (2017). Distinct subtypes of apathy revealed by the apathy motivation index. PLoS ONE.

[37] Creswell KG, Wright AG, Flory JD, Skrzynski CJ, Manuck SB (2018). Multidimensional assessment of impulsivity-related measures in relation to externalizing behaviors. Psychol. Med..

[38] Marin RS, Biedrzycki RC, Firinciogullari S (1991). Reliability and validity of the Apathy Evaluation Scale. Psychiatry Res..

[39] Sockeel P (2006). The lille apathy rating scale (lars), a new instrument for detecting and quantifying apathy: validation in parkinson’s disease. J. Neurol. Neurosurg. Psychiatry.

[40] Patton JH, Stanford MS, Barratt ES (1995). Factor structure of the Barratt impulsiveness scale. J. Clin. Psychol..

[41] Lynam DR, Smith GT, Whiteside SP, Cyders MA (2006). The UPPS-P: Assessing Five Personality Pathways to Impulsive behavior.

[42] Gillan CM, Kosinski M, Whelan R, Phelps EA, Daw ND (2016). Characterizing a psychiatric symptom dimension related to deficits in goal-directed control. eLife.

[43] Patzelt EH, Kool W, Millner AJ, Gershman SJ (2019). Incentives boost model-based control across a range of severity on several psychiatric constructs. Biol. Psychiatry.

[44] Rouault M, Seow T, Gillan CM, Fleming SM (2018). Psychiatric symptom dimensions are associated with dissociable shifts in metacognition but not task performance. Biol. Psychiatry.

[45] Seow TXF, Gillan CM (2020). Transdiagnostic phenotyping reveals a host of metacognitive deficits implicated in compulsivity. Sci. Rep..

[46] Kant R, Duffy JD, Pivovarnik A (1998). Prevalence of apathy following head injury. Brain Inj..

[47] Stanford MS (2009). Fifty years of the Barratt Impulsiveness Scale: an update and review. Pers. Individ. Differ..

[48] Huys QJM (2012). Bonsai trees in your head: how the pavlovian system sculpts goal-directed choices by pruning decision trees. PLOS Comput. Biol..

[49] Reber J (2017). Selective impairment of goal-directed decision-making following lesions to the human ventromedial prefrontal cortex. Brain.

[50] Gillan CM (2020). Comparison of the association between goal-directed planning and self-reported compulsivity vs obsessive-compulsive disorder diagnosis. JAMA Psychiatry.

[51] Lüscher C, Robbins TW, Everitt BJ (2020). The transition to compulsion in addiction. Nat. Rev. Neurosci..

[52] Schreiner DC, Renteria R, Gremel CM (2020). Fractionating the all-or-nothing definition of goal-directed and habitual decision-making. J. Neurosci. Res..

[53] Redgrave P (2010). Goal-directed and habitual control in the basal ganglia: implications for parkinson’s disease. Nat. Rev. Neurosci..

[54] Rangel A, Camerer C, Montague PR (2008). A framework for studying the neurobiology of value-based decision making. Nat. Rev. Neurosci..

[55] Levy DJ, Glimcher PW (2012). The root of all value: a neural common currency for choice. Curr. Opin. Neurobiol..

[56] Bartra O, McGuire JT, Kable JW (2013). The valuation system: a coordinate-based meta-analysis of bold fmri experiments examining neural correlates of subjective value. NeuroImage.

[57] Izuma K, Saito DN, Sadato N (2008). Processing of social and monetary rewards in the human striatum. Neuron.

[58] Piva M (2019). The dorsomedial prefrontal cortex computes task-invariant relative subjective value for self and other. eLife.

[59] Ledoux JE (1989). Cognitive-emotional interactions in the brain. Cogn. Emot..

[60] Dalgleish T (2004). The emotional brain. Nat. Rev. Neurosci..

[61] Shamay-Tsoory SG (2011). The neural bases for empathy. Neuroscience.

[62] Lockwood PL, Ang Y-S, Husain M, Crockett MJ (2017). Individual differences in empathy are associated with apathy-motivation. Sci. Rep..

[63] Levy R, Dubois B (2005). Apathy and the functional anatomy of the prefrontal cortex-basal ganglia circuits. Cereb. Cortex.

[64] Radakovic R (2017). Multidimensional apathy and executive dysfunction in amyotrophic lateral sclerosis. Cortex.

[65] Buckholtz JW (2010). Dopaminergic network differences in human impulsivity. Science.

[66] Baggio HC (2015). Resting-state frontostriatal functional connectivity in Parkinson’s disease-related apathy. Mov. Disord..

[67] Bonnelle V, Manohar S, Behrens T, Husain M (2015). Individual differences in premotor brain systems underlie behavioral apathy. Cereb. Cortex.

[68] Lockwood PL (2017). Prosocial apathy for helping others when effort is required. Nat. Human Behav..

[69] Ang Y-S (2018). Dopamine modulates option generation for behavior. Curr. Biol..

[70] Hammes J (2019). Dopamine metabolism of the nucleus accumbens and fronto-striatal connectivity modulate impulse control. Brain.

[71] Steeves TD (2009). Increased striatal dopamine release in Parkinsonian patients with pathological gambling: A [11C] raclopride PET study. Brain.

[72] Joutsa J (2012). Mesolimbic dopamine release is linked to symptom severity in pathological gambling. NeuroImage.

[73] Raven J (2000). The raven’s progressive matrices: change and stability over culture and time. Cogn. Psychol..

[74] R Core Team. *R: A Language and Environment for Statistical Computing*. R Foundation for Statistical Computing, Vienna, Austria (2018).

[75] Hittner JB, May K, Silver NC (2003). A monte carlo evaluation of tests for comparing dependent correlations. J. Gen. Psychol..

[76] Horn JL (1965). A rationale and test for the number of factors in factor analysis. Psychometrika.

