# Supplementary material for: The relationship between apathy and impulsivity in large population samples
Source: Sci Rep. 2021 Mar 1;11:4830. doi: 10.1038/s41598-021-84364-w (PMC7921138; doi:10.1038/s41598-021-84364-w)
Supplement: Supplementary file 1 — Supplementary Information. [file 41598_2021_84364_MOESM1_ESM.pdf]

Supplementary Information

The relationship between apathy and impulsivity in large population samples

Pierre Petitet, Jacqueline Scholl, Bahaaeddin Attaallah, Daniel Drew, Sanjay Manohar, and Masud Husain

Additional methods

Confirmatory Factor Analyses of the four questionnaires

A Confirmatory Factor Analyses (CFA) was run for each questionnaire to check the validity of their factor structure (Tables S3-S6). These analyses were performed using the R package *lavaan* (`http://lavaan.org`), and using the "group" option to fit the same model to all datasets including a given questionnaire. Similar to previous work[34], the guidelines used to assess model fit were:

- χ^2/df < 3 suggests good fit;
- root mean square error of approximation (RMSEA) < 0.08 acceptable, < 0.05 excellent;
- comparative fit index (CFI) > 0.90 acceptable, > 0.95 excellent;
- non-normative fit index (NFI) $> .95$ good;
- standard root mean square residual (SRMR) < 0.10 acceptable, < 0.05 good

As illustrated in Table S1, most questionnaires failed to meet the standard criteria for good reproduciblity of their original factor structure. The sub-components of the AMI and UPPS-P were slightly more robust than those of the AES and BIS, but were nevertheless still only marginally acceptable.

Additional results

Online participants are more apathetic than laboratory ones

Fig. S2 compare the AMI sub-scale scores of online participants (Dataset 5) versus laboratory participants (Dataset 6). As a group, laboratory participants scored significantly lower on both the behavioural and social sub-scales (both $p < 10^{-7}$).

Identical AES:BIS-11 structure in all four open datasets

The pooled sub-scale correlation matrix of Datasets 1-4 (Fig. 2) was representative of the structure found in individual datasets.

Partial correlations between AES, BIS-11 and UPPS-P

Partial correlations were used to explore in more detail why the apathy and impulsivity factors became negatively correlated when adding the UPPS-P data to the sub-scale correlation matrix in Dataset 3. As reported in the main text, pairwise correlations all showed positive associations between the questionnaire total scores (Fig. 3). However, when controlling for the BIS-11 (partial correlation), the relationship between AES and UPPS-P became negative ($r_{(806)} = -0.10$, $p = 0.005$). By contrast, the other two relationships remained positive (BIS-11 vs. UPPS-P: $r_{(806)} = 0.60$, $p < 10^{-80}$; BIS-11 vs. AES: $r_{(806)} = 0.55$, $p < 10-64$) when controlling for the other questionnaire.

Independent replication of the factor structure of Dataset 5

The laboratory dataset (Dataset 6; $n = 176$, 89 females; mean age: 24.79, *s.d.*: 4.31) contained AMI and BIS-11 data. These questionnaires were filled out by young healthy volunteers who took part in various task-based behavioural experiments in our laboratory. Once again, the sub-scale correlation matrix showed evidence of both positive (AMI behavioural vs. BIS-11 attention, AMI behavioural vs. BIS-11 non-planning) and negative (AMI social vs. BIS-11 motor) associations. The factor decomposition of this matrix (EFA) yielded a very similar structure to the one observed in Dataset 5 (Fig. S3). That is an "Apathy-Impulsivity overlap" factor, an "Apathy-Impulsivity axis" factor, and an emotional apathy factor.

To formally assess the reproducibility of the factor structure observed in Dataset 5, a CFA was performed (Table S7). Only sub-scales with an original absolute loading greater than 0.2 were included in the latent factors. Model fit indices are summarised bellow:

- $\chi^2(5) = 18.83$

- χ^2/df ratio: 3.77
- RMSEA: 0.13
- CFI: 0.93
- NNFI: 0.80
- SRMR: 0.05

Overall, this shows a good reproducibility of the original factor structure.

In order to provide an overview of the items driving the cross-questionnaire structure reported in Figs. 4 & S3, a 3-factor EFA was performed at the item level on the combined Datasets 5-6 (total $n = 569$ complete observations). This analysis highlighted the same structure as when using sub-scores while revealing the contribution of individual questions.

Additional tables

	AES	AMI	BIS-11	UPPS-P
χ^2	5072.31	844.00	17433.48	8155.19
df	452	260	2412	1642
χ^2/df ratio	11.22	3.25	7.23	4.97
RMSEA	0.11	0.09	0.10	0.07
CFI	0.77	0.80	0.60	0.80
NNFI	0.73	0.77	0.57	0.79
SRMR	0.07	0.09	0.10	0.11

Table S1. Model Fit indices for the questionnaire Confirmatory Factor Analyses. Models were fitted to all datasets including a given questionnaire using the “multiple groups” option. All $\chi^2 ps < 10^{-4}$. RMSEA: root mean square error of approximation; CFI: comparative fit index; NNFI: nonnormative fit index; SRMR = standard root mean square residual

Additional tables (submitted as separate files):

Table S2. Detailed description of the questionnaires used in this paper. See attached.
Table S3. Confirmatory Factor Analysis of the AES questionnaire. See attached.
Table S4. Confirmatory Factor Analysis of the AMI questionnaire. See attached.
Table S5. Confirmatory Factor Analysis of the BIS-11 questionnaire. See attached.
Table S6. Confirmatory Factor Analysis of the UPPS-P questionnaire. See attached.
Table S7. Confirmatory Factor Analysis of the combined AMI/BIS-11 questionnaires. See attached.

Additional figures

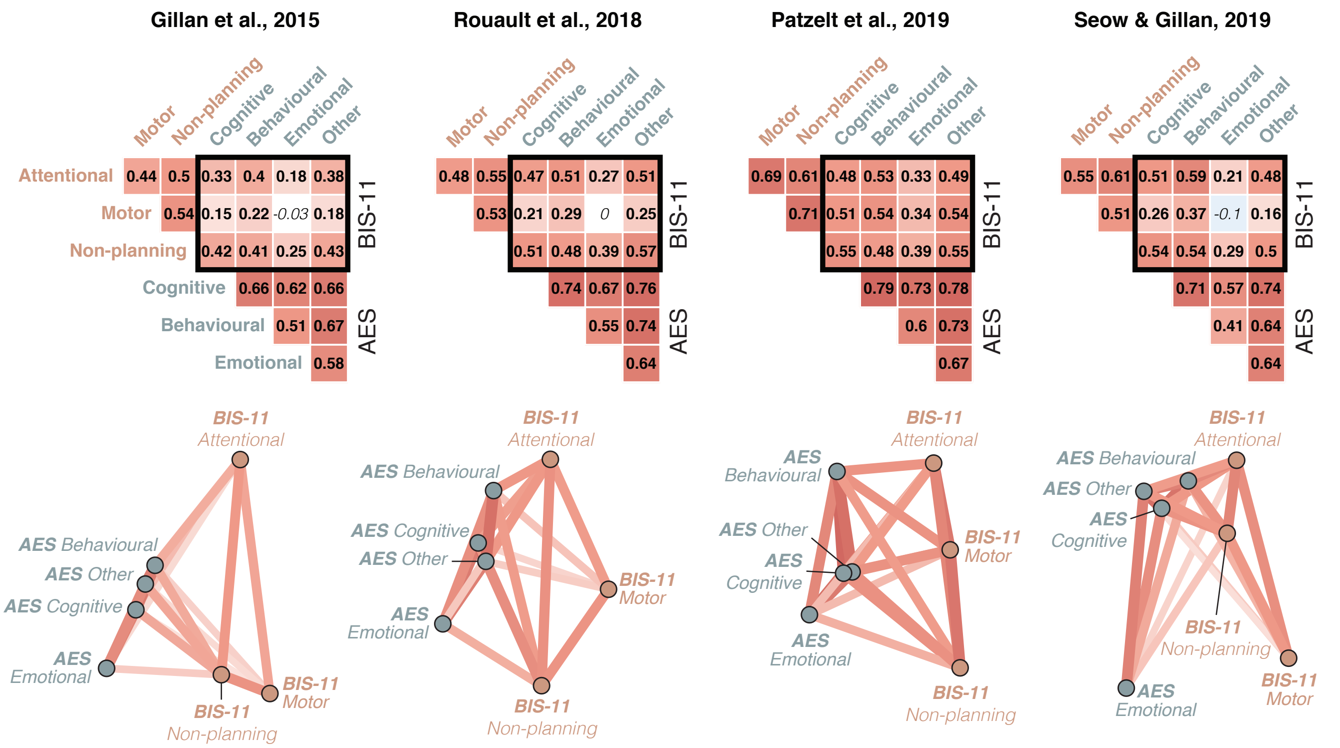

Figure S1. All AES (apathy) and BIS-11 (impulsivity) sub-scales positively correlate in Datasets 1-4. a. The correlation matrix of the sub-scale scores of the AES and BIS-11 questionnaires was dominated by positive correlations. Significant correlations are shown in bold (Bonferroni-corrected significance threshold: $p < 0.002$). In the network plots, sub-scale scores that are more highly correlated appear closer together and are joined by stronger paths. Paths are also coloured by their sign (blue for positive and red for negative). The proximity of the points is determined by multidimensional clustering. Non-significant paths are removed.

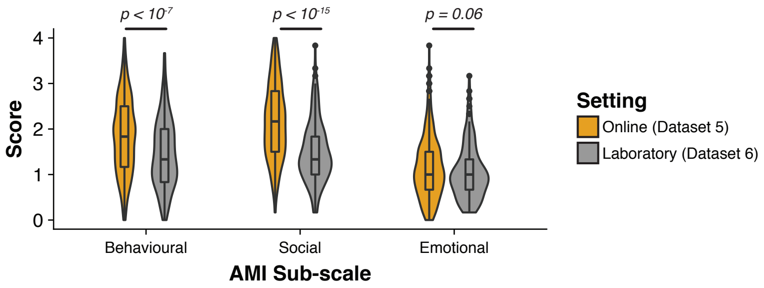

Figure S2. Comparison of AMI sub-scale score in an online versus laboratory setting. Online participants (Dataset 5, $n = 394$) scored significantly higher than laboratory participants (Dataset 6, $n = 176$) on the behavioural and social sub-scales of the AMI questionnaire. Box plots are overlaid on top of violin plots. The lower and upper hinges correspond to the first and third quartiles (the 25th and 75th percentiles). Outliers (values extending outside the hinges for more than 150% of the inter-quartile range) are shown as separate dots. The whiskers extend from the hinges to the most extreme non-outlier data point.

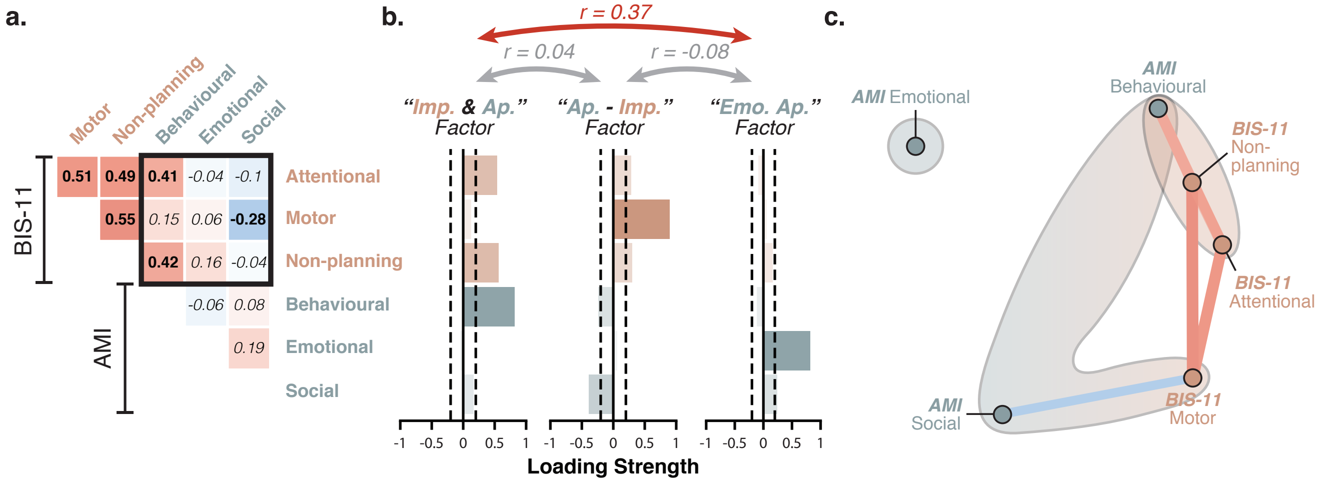

Figure S3. **The factor structure of the combined AMI/BIS-11 replicates across datasets.** **a.** The correlation matrix of the sub-scale scores of the AMI and BIS-11 questionnaires showed positive and negative associations (Datasets 6; $n = 176$). Significant correlations are shown in bold (Bonferroni-corrected significance threshold: $p < 0.003$). The black square highlights cross-questionnaire associations. **b.** Factor decomposition (EFA) of this matrix yielded 3 factors (Apathy-Impulsivity overlap, Apathy-Impulsivity axis, Emotional apathy). This structure was similar to the one observed in Dataset 5 (Fig. 4). **c.** The network plot is shown for visualisation purposes. More highly correlated (positively or negatively) appear closer together and are joined by stronger paths (non-significant paths removed). Paths are also coloured by their sign (red for positive and blue negative). Ellipses are drawn around the sub-scores included in a factor.

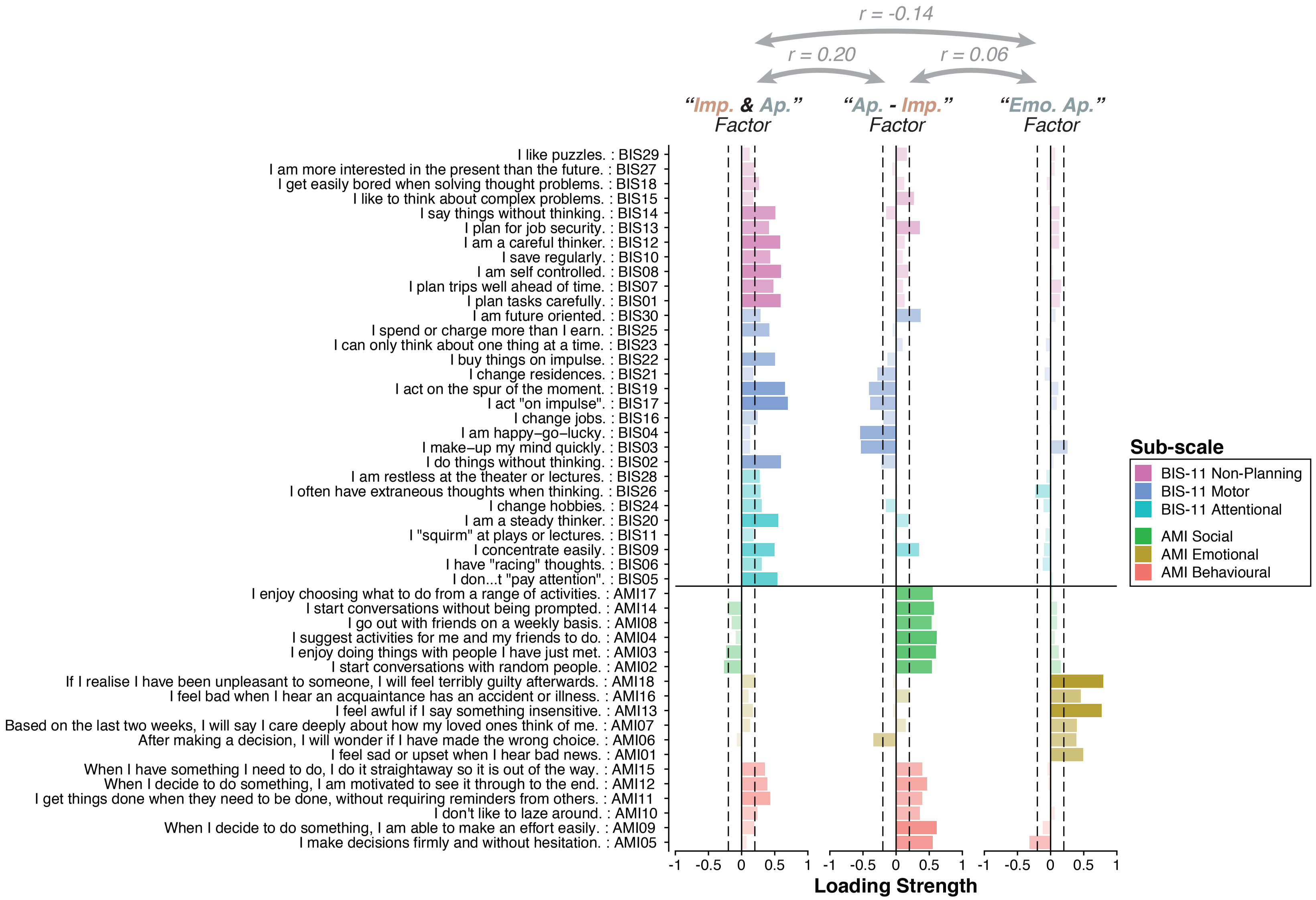

Figure S4. **Item-wise 3-factor EFA of the combined AMI/BIS-11 questionnaires.** The three factors extracted at the item level were similar to those extracted at the sub-score level. The EFA was performed on the combined Datasets 5-6 ($n = 569$ complete observations).
