# Supplementary material for: The relationship between apathy and impulsivity in large population samples
Source: Sci Rep. 2021 Mar 1;11:4830. doi: 10.1038/s41598-021-84364-w (PMC7921138; doi:10.1038/s41598-021-84364-w)
Supplement: Supplementary file 3 — Supplementary Information. [file 41598_2021_84364_MOESM3_ESM.html]

|  |  |  |  |  |  |  |  |  |  |  |  |  |  |  |  |  |
| --- | --- | --- | --- | --- | --- | --- | --- | --- | --- | --- | --- | --- | --- | --- | --- | --- |
|  | Dataset1 | | | | Dataset2 | | | | Dataset3 | | | | Dataset4 | | | |
|  | Estimate | Std. Err. | z | p | Estimate | Std. Err. | z | p | Estimate | Std. Err. | z | p | Estimate | Std. Err. | z | p |
|  | Factor Loadings | | | | | | | | |
| cognitive |
| aes01 | 0.44 | 0.02 | 25.14 | .000 | 0.50 | 0.03 | 14.69 | .000 | 0.62 | 0.02 | 27.07 | .000 | 0.54 | 0.03 | 16.98 | .000 |
| aes03 | 0.41 | 0.02 | 20.20 | .000 | 0.57 | 0.03 | 16.49 | .000 | 0.57 | 0.02 | 24.48 | .000 | 0.49 | 0.03 | 14.36 | .000 |
| aes04 | 0.37 | 0.02 | 17.37 | .000 | 0.46 | 0.04 | 11.56 | .000 | 0.57 | 0.03 | 20.02 | .000 | 0.45 | 0.04 | 11.00 | .000 |
| aes05 | 0.30 | 0.02 | 17.11 | .000 | 0.44 | 0.04 | 12.60 | .000 | 0.56 | 0.03 | 21.69 | .000 | 0.41 | 0.03 | 11.97 | .000 |
| aes08 | 0.42 | 0.02 | 23.11 | .000 | 0.53 | 0.04 | 15.09 | .000 | 0.55 | 0.02 | 22.55 | .000 | 0.45 | 0.03 | 13.88 | .000 |
| aes11 | 0.28 | 0.03 | 9.79 | .000 | 0.42 | 0.04 | 10.29 | .000 | 0.33 | 0.03 | 11.88 | .000 | 0.27 | 0.05 | 5.38 | .000 |
| aes13 | 0.41 | 0.03 | 15.41 | .000 | 0.45 | 0.05 | 9.34 | .000 | 0.54 | 0.03 | 15.85 | .000 | 0.48 | 0.05 | 9.37 | .000 |
| aes16 | 0.49 | 0.02 | 26.79 | .000 | 0.61 | 0.03 | 18.15 | .000 | 0.58 | 0.02 | 24.96 | .000 | 0.47 | 0.03 | 14.95 | .000 |
| behavioural |
| aes02 | 0.53 | 0.02 | 26.01 | .000 | 0.71 | 0.04 | 19.08 | .000 | 0.54 | 0.02 | 23.25 | .000 | 0.50 | 0.03 | 15.87 | .000 |
| aes06 | 0.45 | 0.03 | 17.51 | .000 | 0.49 | 0.03 | 14.04 | .000 | 0.45 | 0.03 | 15.56 | .000 | 0.50 | 0.04 | 11.14 | .000 |
| aes09 | 0.36 | 0.02 | 18.47 | .000 | 0.39 | 0.03 | 11.74 | .000 | 0.52 | 0.02 | 21.47 | .000 | 0.47 | 0.04 | 13.03 | .000 |
| aes10 | 0.20 | 0.02 | 9.47 | .000 | 0.21 | 0.03 | 7.36 | .000 | 0.11 | 0.02 | 4.95 | .000 | 0.22 | 0.04 | 5.00 | .000 |
| aes12 | 0.40 | 0.02 | 16.66 | .000 | 0.48 | 0.04 | 10.99 | .000 | 0.57 | 0.03 | 17.97 | .000 | 0.49 | 0.05 | 10.75 | .000 |
| emotional |
| aes07 | 0.59 | 0.03 | 21.98 | .000 | 0.65 | 0.05 | 14.02 | .000 | 0.64 | 0.03 | 18.58 | .000 | 0.70 | 0.05 | 14.21 | .000 |
| aes14 | 0.35 | 0.02 | 16.97 | .000 | 0.39 | 0.04 | 11.06 | .000 | 0.49 | 0.03 | 16.46 | .000 | 0.43 | 0.04 | 11.53 | .000 |
| other |
| aes15 | 0.29 | 0.02 | 14.51 | .000 | 0.42 | 0.03 | 12.15 | .000 | 0.42 | 0.03 | 16.53 | .000 | 0.34 | 0.03 | 11.07 | .000 |
| aes17 | 0.66 | 0.03 | 25.87 | .000 | 0.76 | 0.04 | 19.02 | .000 | 0.68 | 0.03 | 23.64 | .000 | 0.71 | 0.04 | 18.64 | .000 |
|  | Intercepts | | | | | | | | |
| aes01 | 1.58 | 0.02 | 85.01 | .000 | 1.68 | 0.04 | 46.10 | .000 | 1.71 | 0.03 | 63.59 | .000 | 1.55 | 0.04 | 44.13 | .000 |
| aes03 | 1.62 | 0.02 | 79.22 | .000 | 1.77 | 0.04 | 46.96 | .000 | 1.70 | 0.03 | 63.58 | .000 | 1.58 | 0.04 | 43.17 | .000 |
| aes04 | 1.61 | 0.02 | 76.00 | .000 | 1.88 | 0.04 | 45.97 | .000 | 1.98 | 0.03 | 63.53 | .000 | 1.79 | 0.04 | 43.01 | .000 |
| aes05 | 1.39 | 0.02 | 80.25 | .000 | 1.57 | 0.04 | 42.82 | .000 | 1.74 | 0.03 | 61.03 | .000 | 1.50 | 0.04 | 42.59 | .000 |
| aes08 | 1.53 | 0.02 | 81.45 | .000 | 1.66 | 0.04 | 43.73 | .000 | 1.64 | 0.03 | 60.38 | .000 | 1.48 | 0.03 | 42.56 | .000 |
| aes11 | 2.03 | 0.03 | 74.37 | .000 | 1.75 | 0.04 | 41.95 | .000 | 1.62 | 0.03 | 57.87 | .000 | 1.81 | 0.05 | 37.77 | .000 |
| aes13 | 2.10 | 0.03 | 81.59 | .000 | 2.45 | 0.05 | 50.54 | .000 | 2.38 | 0.04 | 66.36 | .000 | 2.15 | 0.05 | 41.87 | .000 |
| aes16 | 1.61 | 0.02 | 81.94 | .000 | 1.67 | 0.04 | 44.17 | .000 | 1.64 | 0.03 | 61.03 | .000 | 1.45 | 0.03 | 42.78 | .000 |
| aes02 | 1.80 | 0.02 | 85.74 | .000 | 1.83 | 0.04 | 43.91 | .000 | 1.67 | 0.03 | 64.52 | .000 | 1.54 | 0.03 | 46.23 | .000 |
| aes06 | 2.00 | 0.03 | 79.06 | .000 | 1.50 | 0.04 | 41.30 | .000 | 1.60 | 0.03 | 53.65 | .000 | 1.71 | 0.04 | 38.04 | .000 |
| aes09 | 1.64 | 0.02 | 83.61 | .000 | 1.64 | 0.03 | 48.40 | .000 | 1.77 | 0.03 | 67.35 | .000 | 1.63 | 0.04 | 44.25 | .000 |
| aes10 | 1.46 | 0.02 | 72.45 | .000 | 1.24 | 0.03 | 44.87 | .000 | 1.24 | 0.02 | 57.71 | .000 | 1.46 | 0.04 | 35.58 | .000 |
| aes12 | 1.99 | 0.02 | 83.79 | .000 | 2.04 | 0.04 | 46.22 | .000 | 2.09 | 0.03 | 61.89 | .000 | 1.98 | 0.05 | 43.86 | .000 |
| aes07 | 2.25 | 0.02 | 95.79 | .000 | 2.44 | 0.04 | 58.01 | .000 | 2.50 | 0.03 | 74.90 | .000 | 2.27 | 0.05 | 49.45 | .000 |
| aes14 | 1.50 | 0.02 | 78.40 | .000 | 1.60 | 0.03 | 47.49 | .000 | 1.79 | 0.03 | 60.95 | .000 | 1.56 | 0.04 | 44.13 | .000 |
| aes15 | 1.62 | 0.02 | 84.41 | .000 | 1.74 | 0.04 | 48.91 | .000 | 1.70 | 0.03 | 65.55 | .000 | 1.50 | 0.03 | 48.58 | .000 |
| aes17 | 1.86 | 0.02 | 83.47 | .000 | 1.90 | 0.04 | 45.44 | .000 | 1.90 | 0.03 | 63.29 | .000 | 1.72 | 0.04 | 43.71 | .000 |
|  | Residual Variances | | | | | | | | |
| aes01 | 0.29 | 0.01 | 23.69 | .000 | 0.41 | 0.03 | 14.76 | .000 | 0.23 | 0.01 | 17.89 | .000 | 0.25 | 0.02 | 12.77 | .000 |
| aes03 | 0.42 | 0.02 | 24.96 | .000 | 0.38 | 0.03 | 14.36 | .000 | 0.27 | 0.01 | 18.67 | .000 | 0.34 | 0.03 | 13.58 | .000 |
| aes04 | 0.49 | 0.02 | 25.45 | .000 | 0.62 | 0.04 | 15.22 | .000 | 0.49 | 0.03 | 19.49 | .000 | 0.56 | 0.04 | 14.17 | .000 |
| aes05 | 0.34 | 0.01 | 25.49 | .000 | 0.47 | 0.03 | 15.09 | .000 | 0.37 | 0.02 | 19.24 | .000 | 0.38 | 0.03 | 14.03 | .000 |
| aes08 | 0.32 | 0.01 | 24.29 | .000 | 0.43 | 0.03 | 14.68 | .000 | 0.32 | 0.02 | 19.08 | .000 | 0.32 | 0.02 | 13.68 | .000 |
| aes11 | 0.97 | 0.04 | 26.26 | .000 | 0.68 | 0.04 | 15.35 | .000 | 0.55 | 0.03 | 20.20 | .000 | 0.93 | 0.06 | 14.66 | .000 |
| aes13 | 0.77 | 0.03 | 25.72 | .000 | 0.96 | 0.06 | 15.43 | .000 | 0.78 | 0.04 | 19.93 | .000 | 0.92 | 0.06 | 14.36 | .000 |
| aes16 | 0.30 | 0.01 | 23.09 | .000 | 0.33 | 0.02 | 13.86 | .000 | 0.27 | 0.01 | 18.55 | .000 | 0.28 | 0.02 | 13.43 | .000 |
| aes02 | 0.34 | 0.02 | 21.89 | .000 | 0.35 | 0.03 | 11.94 | .000 | 0.27 | 0.01 | 18.00 | .000 | 0.24 | 0.02 | 11.65 | .000 |
| aes06 | 0.70 | 0.03 | 25.46 | .000 | 0.42 | 0.03 | 14.56 | .000 | 0.55 | 0.03 | 19.93 | .000 | 0.63 | 0.05 | 13.74 | .000 |
| aes09 | 0.41 | 0.02 | 25.26 | .000 | 0.42 | 0.03 | 15.04 | .000 | 0.32 | 0.02 | 18.75 | .000 | 0.37 | 0.03 | 13.18 | .000 |
| aes10 | 0.54 | 0.02 | 26.34 | .000 | 0.34 | 0.02 | 15.52 | .000 | 0.38 | 0.02 | 20.45 | .000 | 0.69 | 0.05 | 14.61 | .000 |
| aes12 | 0.63 | 0.02 | 25.61 | .000 | 0.73 | 0.05 | 15.15 | .000 | 0.63 | 0.03 | 19.60 | .000 | 0.65 | 0.05 | 13.83 | .000 |
| aes07 | 0.43 | 0.03 | 16.63 | .000 | 0.47 | 0.05 | 10.05 | .000 | 0.52 | 0.03 | 15.31 | .000 | 0.43 | 0.05 | 8.56 | .000 |
| aes14 | 0.39 | 0.02 | 23.97 | .000 | 0.41 | 0.03 | 14.05 | .000 | 0.48 | 0.03 | 17.69 | .000 | 0.37 | 0.03 | 12.57 | .000 |
| aes15 | 0.44 | 0.02 | 25.63 | .000 | 0.45 | 0.03 | 14.90 | .000 | 0.39 | 0.02 | 19.15 | .000 | 0.30 | 0.02 | 13.98 | .000 |
| aes17 | 0.26 | 0.03 | 10.28 | .000 | 0.29 | 0.04 | 7.94 | .000 | 0.30 | 0.02 | 12.49 | .000 | 0.17 | 0.03 | 5.21 | .000 |
|  | Latent Intercepts | | | | | | | | |
| cognitive | 0.00+ |  |  |  | 0.00+ |  |  |  | 0.00+ |  |  |  | 0.00+ |  |  |  |
| behavioural | 0.00+ |  |  |  | 0.00+ |  |  |  | 0.00+ |  |  |  | 0.00+ |  |  |  |
| emotional | 0.00+ |  |  |  | 0.00+ |  |  |  | 0.00+ |  |  |  | 0.00+ |  |  |  |
| other | 0.00+ |  |  |  | 0.00+ |  |  |  | 0.00+ |  |  |  | 0.00+ |  |  |  |
|  | Latent Variances | | | | | | | | |
| cognitive | 1.00+ |  |  |  | 1.00+ |  |  |  | 1.00+ |  |  |  | 1.00+ |  |  |  |
| behavioural | 1.00+ |  |  |  | 1.00+ |  |  |  | 1.00+ |  |  |  | 1.00+ |  |  |  |
| emotional | 1.00+ |  |  |  | 1.00+ |  |  |  | 1.00+ |  |  |  | 1.00+ |  |  |  |
| other | 1.00+ |  |  |  | 1.00+ |  |  |  | 1.00+ |  |  |  | 1.00+ |  |  |  |
|  | Latent Covariances | | | | | | | | |
| cognitive w/behavioural | 0.97 | 0.02 | 51.52 | .000 | 0.98 | 0.02 | 45.57 | .000 | 1.01 | 0.01 | 79.46 | .000 | 0.96 | 0.03 | 37.65 | .000 |
| cognitive w/emotional | 0.99 | 0.03 | 31.99 | .000 | 0.96 | 0.04 | 21.47 | .000 | 1.02 | 0.03 | 34.54 | .000 | 0.82 | 0.04 | 18.34 | .000 |
| cognitive w/other | 0.94 | 0.03 | 33.09 | .000 | 0.99 | 0.03 | 32.34 | .000 | 1.03 | 0.02 | 45.77 | .000 | 0.93 | 0.03 | 28.01 | .000 |
| behavioural w/emotional | 0.94 | 0.04 | 26.13 | .000 | 0.82 | 0.05 | 15.86 | .000 | 0.98 | 0.03 | 28.36 | .000 | 0.73 | 0.05 | 13.41 | .000 |
| behavioural w/other | 1.06 | 0.03 | 32.48 | .000 | 1.01 | 0.03 | 29.46 | .000 | 1.06 | 0.03 | 40.26 | .000 | 0.86 | 0.04 | 21.44 | .000 |
| emotional w/other | 0.99 | 0.04 | 23.82 | .000 | 1.01 | 0.05 | 18.87 | .000 | 1.10 | 0.04 | 27.37 | .000 | 0.91 | 0.05 | 18.37 | .000 |
|  | Fit Indices | | | | | | | | |
| χ2 | 5072.31(452) |  |  | .000 |  |  |  |  |  |  |  |  |  |  |  |  |
| DF | 452.00 |  |  |  |  |  |  |  |  |  |  |  |  |  |  |  |
| RMSEA | 0.11 |  |  |  |  |  |  |  |  |  |  |  |  |  |  |  |
| CFI | 0.77 |  |  |  |  |  |  |  |  |  |  |  |  |  |  |  |
| NNFI | 0.73 |  |  |  |  |  |  |  |  |  |  |  |  |  |  |  |
| SRMR | 0.07 |  |  |  |  |  |  |  |  |  |  |  |  |  |  |  |
| \_BOML10\_+Fixed parameter |

  
