# Supplementary material for: The relationship between apathy and impulsivity in large population samples
Source: Sci Rep. 2021 Mar 1;11:4830. doi: 10.1038/s41598-021-84364-w (PMC7921138; doi:10.1038/s41598-021-84364-w)
Supplement: Supplementary file 4 — Supplementary Information. [file 41598_2021_84364_MOESM4_ESM.html]

|  |  |  |  |  |  |  |  |  |
| --- | --- | --- | --- | --- | --- | --- | --- | --- |
|  | Dataset5 | | | | Dataset6 | | | |
|  | Estimate | Std. Err. | z | p | Estimate | Std. Err. | z | p |
|  | Factor Loadings | | | | | | | |
| behavioural |
| ami05 | 0.68 | 0.06 | 11.45 | .000 | 0.42 | 0.09 | 4.84 | .000 |
| ami09 | 0.74 | 0.05 | 13.93 | .000 | 0.57 | 0.07 | 8.32 | .000 |
| ami10 | 0.57 | 0.07 | 8.79 | .000 | 0.59 | 0.09 | 6.31 | .000 |
| ami11 | 0.86 | 0.06 | 14.68 | .000 | 0.89 | 0.08 | 11.60 | .000 |
| ami12 | 0.77 | 0.05 | 15.11 | .000 | 0.74 | 0.06 | 12.09 | .000 |
| ami15 | 0.77 | 0.06 | 13.35 | .000 | 0.70 | 0.08 | 8.71 | .000 |
| ami13 | 0.04 | 0.05 | 0.78 | .434 | -0.07 | 0.07 | -0.99 | .324 |
| ami16 | 0.19 | 0.05 | 4.07 | .000 | 0.09 | 0.07 | 1.30 | .192 |
| emotional |
| ami01 | 0.43 | 0.05 | 9.29 | .000 | 0.48 | 0.08 | 6.02 | .000 |
| ami06 | 0.33 | 0.06 | 5.46 | .000 | 0.35 | 0.09 | 3.96 | .000 |
| ami07 | 0.42 | 0.06 | 7.38 | .000 | 0.40 | 0.09 | 4.58 | .000 |
| ami13 | 0.96 | 0.06 | 16.99 | .000 | 0.74 | 0.07 | 9.98 | .000 |
| ami16 | 0.45 | 0.05 | 9.60 | .000 | 0.31 | 0.07 | 4.34 | .000 |
| ami18 | 0.91 | 0.05 | 18.54 | .000 | 0.63 | 0.06 | 9.92 | .000 |
| social |
| ami02 | 0.69 | 0.06 | 12.04 | .000 | 0.82 | 0.09 | 9.61 | .000 |
| ami03 | 0.68 | 0.05 | 12.44 | .000 | 0.63 | 0.07 | 8.41 | .000 |
| ami04 | 0.85 | 0.06 | 14.46 | .000 | 0.49 | 0.08 | 6.29 | .000 |
| ami08 | 0.76 | 0.07 | 10.29 | .000 | 0.39 | 0.09 | 4.22 | .000 |
| ami14 | 0.80 | 0.06 | 13.84 | .000 | 0.67 | 0.08 | 8.50 | .000 |
| ami17 | 0.59 | 0.05 | 11.61 | .000 | 0.40 | 0.08 | 5.29 | .000 |
|  | Intercepts | | | | | | | |
| ami05 | 2.12 | 0.06 | 35.82 | .000 | 1.95 | 0.08 | 23.40 | .000 |
| ami09 | 1.72 | 0.06 | 31.03 | .000 | 1.19 | 0.07 | 16.83 | .000 |
| ami10 | 2.31 | 0.06 | 36.90 | .000 | 1.60 | 0.09 | 17.43 | .000 |
| ami11 | 1.58 | 0.06 | 25.69 | .000 | 1.14 | 0.08 | 13.50 | .000 |
| ami12 | 1.27 | 0.05 | 23.57 | .000 | 1.02 | 0.07 | 14.99 | .000 |
| ami15 | 2.11 | 0.06 | 35.73 | .000 | 1.89 | 0.08 | 22.90 | .000 |
| ami13 | 1.23 | 0.06 | 20.35 | .000 | 0.86 | 0.07 | 11.73 | .000 |
| ami16 | 0.94 | 0.05 | 20.39 | .000 | 0.72 | 0.06 | 11.08 | .000 |
| ami01 | 1.16 | 0.05 | 25.59 | .000 | 1.22 | 0.07 | 16.40 | .000 |
| ami06 | 1.45 | 0.06 | 25.41 | .000 | 1.76 | 0.08 | 22.00 | .000 |
| ami07 | 1.13 | 0.05 | 20.71 | .000 | 1.04 | 0.08 | 13.04 | .000 |
| ami18 | 1.00 | 0.05 | 18.85 | .000 | 0.69 | 0.06 | 11.06 | .000 |
| ami02 | 2.88 | 0.06 | 49.93 | .000 | 2.25 | 0.08 | 26.51 | .000 |
| ami03 | 2.49 | 0.05 | 45.51 | .000 | 1.71 | 0.07 | 23.48 | .000 |
| ami04 | 1.77 | 0.06 | 29.05 | .000 | 1.13 | 0.07 | 15.56 | .000 |
| ami08 | 2.39 | 0.07 | 33.35 | .000 | 1.01 | 0.08 | 12.06 | .000 |
| ami14 | 2.10 | 0.06 | 35.44 | .000 | 1.58 | 0.08 | 20.50 | .000 |
| ami17 | 1.32 | 0.05 | 26.17 | .000 | 1.23 | 0.07 | 17.55 | .000 |
|  | Residual Variances | | | | | | | |
| ami05 | 0.92 | 0.07 | 12.55 | .000 | 1.04 | 0.12 | 9.06 | .000 |
| ami09 | 0.66 | 0.06 | 11.50 | .000 | 0.54 | 0.07 | 8.31 | .000 |
| ami10 | 1.22 | 0.09 | 13.24 | .000 | 1.13 | 0.13 | 8.83 | .000 |
| ami11 | 0.75 | 0.07 | 11.07 | .000 | 0.46 | 0.07 | 6.23 | .000 |
| ami12 | 0.55 | 0.05 | 10.79 | .000 | 0.26 | 0.05 | 5.68 | .000 |
| ami15 | 0.78 | 0.07 | 11.79 | .000 | 0.71 | 0.09 | 8.17 | .000 |
| ami13 | 0.52 | 0.06 | 8.15 | .000 | 0.38 | 0.07 | 5.08 | .000 |
| ami16 | 0.61 | 0.05 | 13.05 | .000 | 0.64 | 0.07 | 8.95 | .000 |
| ami01 | 0.62 | 0.05 | 13.26 | .000 | 0.75 | 0.09 | 8.55 | .000 |
| ami06 | 1.17 | 0.08 | 13.80 | .000 | 1.00 | 0.11 | 9.04 | .000 |
| ami07 | 1.00 | 0.07 | 13.58 | .000 | 0.95 | 0.11 | 8.92 | .000 |
| ami18 | 0.29 | 0.05 | 5.86 | .000 | 0.28 | 0.05 | 5.21 | .000 |
| ami02 | 0.82 | 0.07 | 12.09 | .000 | 0.59 | 0.10 | 6.07 | .000 |
| ami03 | 0.72 | 0.06 | 11.91 | .000 | 0.53 | 0.07 | 7.24 | .000 |
| ami04 | 0.74 | 0.07 | 10.76 | .000 | 0.69 | 0.08 | 8.39 | .000 |
| ami08 | 1.45 | 0.11 | 12.73 | .000 | 1.07 | 0.12 | 8.96 | .000 |
| ami14 | 0.75 | 0.07 | 11.17 | .000 | 0.59 | 0.08 | 7.17 | .000 |
| ami17 | 0.65 | 0.05 | 12.27 | .000 | 0.70 | 0.08 | 8.71 | .000 |
|  | Latent Intercepts | | | | | | | |
| behavioural | 0.00+ |  |  |  | 0.00+ |  |  |  |
| emotional | 0.00+ |  |  |  | 0.00+ |  |  |  |
| social | 0.00+ |  |  |  | 0.00+ |  |  |  |
|  | Latent Variances | | | | | | | |
| behavioural | 1.00+ |  |  |  | 1.00+ |  |  |  |
| emotional | 1.00+ |  |  |  | 1.00+ |  |  |  |
| social | 1.00+ |  |  |  | 1.00+ |  |  |  |
|  | Latent Covariances | | | | | | | |
| behavioural w/emotional | -0.08 | 0.06 | -1.19 | .233 | 0.03 | 0.10 | 0.34 | .732 |
| behavioural w/social | 0.52 | 0.05 | 10.70 | .000 | 0.07 | 0.09 | 0.72 | .473 |
| emotional w/social | 0.04 | 0.06 | 0.68 | .493 | 0.22 | 0.09 | 2.34 | .019 |
|  | Fit Indices | | | | | | | |
| χ2 | 844.00(260) |  |  | .000 |  |  |  |  |
| DF | 260.00 |  |  |  |  |  |  |  |
| RMSEA | 0.09 |  |  |  |  |  |  |  |
| CFI | 0.80 |  |  |  |  |  |  |  |
| NNFI | 0.77 |  |  |  |  |  |  |  |
| SRMR | 0.09 |  |  |  |  |  |  |  |
| +Fixed parameter | | | | | | | | |

  
