# Supplementary material for: The relationship between apathy and impulsivity in large population samples
Source: Sci Rep. 2021 Mar 1;11:4830. doi: 10.1038/s41598-021-84364-w (PMC7921138; doi:10.1038/s41598-021-84364-w)
Supplement: Supplementary file 5 — Supplementary Information. [file 41598_2021_84364_MOESM5_ESM.html]

|  |  |  |  |  |  |  |  |  |  |  |  |  |  |  |  |  |  |  |  |  |  |  |  |  |
| --- | --- | --- | --- | --- | --- | --- | --- | --- | --- | --- | --- | --- | --- | --- | --- | --- | --- | --- | --- | --- | --- | --- | --- | --- |
|  | Dataset1 | | | | Dataset2 | | | | Dataset3 | | | | Dataset4 | | | | Dataset5 | | | | Dataset6 | | | |
|  | Estimate | Std. Err. | z | p | Estimate | Std. Err. | z | p | Estimate | Std. Err. | z | p | Estimate | Std. Err. | z | p | Estimate | Std. Err. | z | p | Estimate | Std. Err. | z | p |
|  | Factor Loadings | | | | | | | | |
| attentional |
| bis05 | 0.49 | 0.02 | 22.82 | .000 | 0.40 | 0.03 | 13.42 | .000 | 0.35 | 0.02 | 18.45 | .000 | 0.48 | 0.03 | 13.93 | .000 | 0.38 | 0.04 | 10.35 | .000 | 0.52 | 0.05 | 9.39 | .000 |
| bis06 | 0.52 | 0.03 | 19.76 | .000 | 0.59 | 0.04 | 14.30 | .000 | 0.41 | 0.03 | 14.45 | .000 | 0.50 | 0.04 | 11.40 | .000 | 0.28 | 0.05 | 5.72 | .000 | 0.42 | 0.07 | 6.26 | .000 |
| bis09 | 0.57 | 0.02 | 24.37 | .000 | 0.66 | 0.04 | 16.65 | .000 | 0.66 | 0.03 | 24.20 | .000 | 0.61 | 0.04 | 15.25 | .000 | 0.62 | 0.04 | 14.62 | .000 | 0.41 | 0.06 | 6.96 | .000 |
| bis11 | 0.52 | 0.03 | 19.55 | .000 | 0.51 | 0.04 | 11.99 | .000 | 0.47 | 0.03 | 15.43 | .000 | 0.54 | 0.05 | 11.69 | .000 | 0.12 | 0.05 | 2.57 | .010 | 0.44 | 0.07 | 6.36 | .000 |
| bis20 | 0.42 | 0.02 | 19.16 | .000 | 0.50 | 0.04 | 14.03 | .000 | 0.53 | 0.02 | 22.62 | .000 | 0.51 | 0.04 | 14.16 | .000 | 0.50 | 0.04 | 13.71 | .000 | 0.38 | 0.05 | 7.11 | .000 |
| bis24 | 0.26 | 0.02 | 11.30 | .000 | 0.29 | 0.04 | 7.30 | .000 | 0.30 | 0.02 | 12.09 | .000 | 0.38 | 0.04 | 9.80 | .000 | 0.15 | 0.04 | 3.42 | .001 | 0.24 | 0.07 | 3.52 | .000 |
| bis26 | 0.45 | 0.03 | 17.86 | .000 | 0.54 | 0.04 | 12.93 | .000 | 0.44 | 0.03 | 16.01 | .000 | 0.52 | 0.04 | 12.70 | .000 | 0.21 | 0.05 | 4.61 | .000 | 0.41 | 0.07 | 6.13 | .000 |
| bis28 | 0.51 | 0.03 | 20.36 | .000 | 0.50 | 0.04 | 11.51 | .000 | 0.51 | 0.03 | 16.34 | .000 | 0.62 | 0.04 | 13.96 | .000 | 0.19 | 0.05 | 3.90 | .000 | 0.56 | 0.07 | 8.51 | .000 |
| motor |
| bis02 | 0.54 | 0.02 | 26.62 | .000 | 0.53 | 0.03 | 15.15 | .000 | 0.54 | 0.02 | 24.93 | .000 | 0.55 | 0.04 | 15.68 | .000 | 0.44 | 0.04 | 10.78 | .000 | 0.43 | 0.06 | 7.17 | .000 |
| bis03 | 0.04 | 0.03 | 1.53 | .127 | 0.01 | 0.04 | 0.16 | .876 | -0.06 | 0.03 | -1.91 | .056 | -0.09 | 0.05 | -1.85 | .064 | 0.19 | 0.05 | 3.99 | .000 | 0.20 | 0.07 | 2.83 | .005 |
| bis04 | -0.00 | 0.03 | -0.01 | .994 | -0.03 | 0.05 | -0.61 | .543 | -0.04 | 0.04 | -1.12 | .265 | -0.02 | 0.05 | -0.35 | .726 | 0.12 | 0.05 | 2.34 | .020 | 0.34 | 0.07 | 4.65 | .000 |
| bis16 | 0.27 | 0.02 | 12.22 | .000 | 0.47 | 0.04 | 11.17 | .000 | 0.26 | 0.02 | 10.89 | .000 | 0.40 | 0.04 | 10.29 | .000 | 0.23 | 0.04 | 5.32 | .000 | 0.27 | 0.07 | 4.09 | .000 |
| bis17 | 0.60 | 0.02 | 31.66 | .000 | 0.68 | 0.03 | 20.75 | .000 | 0.58 | 0.02 | 28.56 | .000 | 0.59 | 0.03 | 18.18 | .000 | 0.63 | 0.03 | 18.77 | .000 | 0.64 | 0.05 | 12.04 | .000 |
| bis19 | 0.56 | 0.02 | 28.25 | .000 | 0.59 | 0.03 | 19.28 | .000 | 0.54 | 0.02 | 24.53 | .000 | 0.56 | 0.04 | 15.88 | .000 | 0.58 | 0.04 | 16.23 | .000 | 0.55 | 0.05 | 10.80 | .000 |
| bis21 | 0.19 | 0.02 | 9.07 | .000 | 0.32 | 0.04 | 8.77 | .000 | 0.18 | 0.02 | 8.46 | .000 | 0.46 | 0.04 | 12.52 | .000 | 0.19 | 0.04 | 5.11 | .000 | 0.09 | 0.07 | 1.29 | .196 |
| bis22 | 0.48 | 0.02 | 22.94 | .000 | 0.50 | 0.04 | 13.05 | .000 | 0.48 | 0.02 | 20.54 | .000 | 0.50 | 0.03 | 14.34 | .000 | 0.49 | 0.04 | 11.40 | .000 | 0.42 | 0.06 | 6.52 | .000 |
| bis23 | 0.06 | 0.02 | 2.66 | .008 | 0.13 | 0.04 | 3.53 | .000 | 0.23 | 0.03 | 7.61 | .000 | 0.36 | 0.05 | 7.85 | .000 | -0.00 | 0.05 | -0.06 | .949 | -0.05 | 0.07 | -0.67 | .503 |
| bis25 | 0.47 | 0.02 | 19.08 | .000 | 0.49 | 0.04 | 11.55 | .000 | 0.40 | 0.03 | 16.03 | .000 | 0.60 | 0.04 | 15.15 | .000 | 0.30 | 0.04 | 6.70 | .000 | 0.38 | 0.07 | 5.54 | .000 |
| bis30 | 0.25 | 0.02 | 9.91 | .000 | 0.24 | 0.04 | 5.70 | .000 | 0.28 | 0.03 | 8.59 | .000 | 0.12 | 0.04 | 2.78 | .005 | 0.06 | 0.05 | 1.18 | .236 | 0.26 | 0.07 | 3.79 | .000 |
| nonplanning |
| bis01 | 0.53 | 0.02 | 23.87 | .000 | 0.64 | 0.04 | 18.03 | .000 | 0.59 | 0.02 | 24.62 | .000 | 0.50 | 0.04 | 13.74 | .000 | 0.58 | 0.04 | 14.12 | .000 | 0.55 | 0.06 | 9.37 | .000 |
| bis07 | 0.49 | 0.03 | 18.28 | .000 | 0.56 | 0.04 | 13.49 | .000 | 0.56 | 0.03 | 19.80 | .000 | 0.54 | 0.04 | 12.99 | .000 | 0.53 | 0.05 | 11.18 | .000 | 0.50 | 0.07 | 7.59 | .000 |
| bis08 | 0.47 | 0.02 | 21.74 | .000 | 0.58 | 0.04 | 16.63 | .000 | 0.55 | 0.02 | 22.64 | .000 | 0.56 | 0.04 | 15.97 | .000 | 0.56 | 0.04 | 14.11 | .000 | 0.52 | 0.06 | 8.90 | .000 |
| bis10 | 0.52 | 0.03 | 18.00 | .000 | 0.52 | 0.05 | 10.74 | .000 | 0.53 | 0.03 | 15.42 | .000 | 0.49 | 0.05 | 10.04 | .000 | 0.49 | 0.05 | 9.57 | .000 | 0.43 | 0.08 | 5.59 | .000 |
| bis12 | 0.47 | 0.02 | 23.71 | .000 | 0.51 | 0.03 | 15.89 | .000 | 0.57 | 0.02 | 24.93 | .000 | 0.55 | 0.03 | 16.95 | .000 | 0.54 | 0.04 | 14.59 | .000 | 0.49 | 0.05 | 9.49 | .000 |
| bis13 | 0.55 | 0.03 | 19.42 | .000 | 0.71 | 0.04 | 16.31 | .000 | 0.62 | 0.03 | 19.56 | .000 | 0.55 | 0.05 | 11.41 | .000 | 0.57 | 0.05 | 11.51 | .000 | 0.48 | 0.07 | 6.93 | .000 |
| bis14 | 0.43 | 0.02 | 19.26 | .000 | 0.40 | 0.04 | 10.17 | .000 | 0.35 | 0.03 | 13.76 | .000 | 0.44 | 0.04 | 11.22 | .000 | 0.22 | 0.04 | 5.22 | .000 | 0.42 | 0.06 | 7.24 | .000 |
| bis15 | 0.22 | 0.03 | 8.51 | .000 | 0.24 | 0.05 | 5.18 | .000 | 0.34 | 0.03 | 10.30 | .000 | 0.36 | 0.05 | 7.27 | .000 | 0.36 | 0.05 | 7.50 | .000 | 0.17 | 0.06 | 2.78 | .005 |
| bis18 | 0.31 | 0.02 | 13.18 | .000 | 0.35 | 0.04 | 8.49 | .000 | 0.40 | 0.03 | 14.88 | .000 | 0.46 | 0.04 | 11.34 | .000 | 0.27 | 0.04 | 6.42 | .000 | 0.11 | 0.06 | 1.87 | .061 |
| bis27 | 0.14 | 0.02 | 5.97 | .000 | 0.19 | 0.04 | 4.55 | .000 | 0.24 | 0.03 | 7.99 | .000 | 0.21 | 0.04 | 4.92 | .000 | 0.07 | 0.04 | 1.50 | .133 | 0.21 | 0.07 | 3.03 | .002 |
| bis29 | 0.22 | 0.03 | 8.26 | .000 | 0.16 | 0.05 | 3.32 | .001 | 0.24 | 0.03 | 7.39 | .000 | 0.30 | 0.05 | 6.30 | .000 | 0.24 | 0.05 | 4.97 | .000 | 0.08 | 0.07 | 1.14 | .256 |
|  | Intercepts | | | | | | | | |
| bis05 | 1.77 | 0.02 | 82.87 | .000 | 1.36 | 0.03 | 44.76 | .000 | 1.31 | 0.02 | 66.16 | .000 | 1.49 | 0.04 | 41.14 | .000 | 1.69 | 0.04 | 46.72 | .000 | 1.82 | 0.06 | 32.17 | .000 |
| bis06 | 2.17 | 0.03 | 84.09 | .000 | 1.97 | 0.04 | 46.23 | .000 | 1.78 | 0.03 | 62.15 | .000 | 1.88 | 0.04 | 42.21 | .000 | 2.24 | 0.05 | 48.58 | .000 | 2.42 | 0.07 | 36.95 | .000 |
| bis09 | 2.21 | 0.02 | 93.31 | .000 | 2.26 | 0.04 | 53.73 | .000 | 2.12 | 0.03 | 70.43 | .000 | 1.91 | 0.04 | 44.81 | .000 | 2.38 | 0.04 | 54.66 | .000 | 2.27 | 0.06 | 39.04 | .000 |
| bis11 | 2.06 | 0.03 | 80.01 | .000 | 1.90 | 0.04 | 44.85 | .000 | 1.78 | 0.03 | 57.64 | .000 | 1.98 | 0.05 | 42.17 | .000 | 1.91 | 0.04 | 43.92 | .000 | 2.03 | 0.07 | 30.72 | .000 |
| bis20 | 1.98 | 0.02 | 93.90 | .000 | 2.04 | 0.04 | 55.84 | .000 | 1.92 | 0.03 | 74.84 | .000 | 1.77 | 0.04 | 46.83 | .000 | 2.19 | 0.04 | 58.89 | .000 | 2.14 | 0.05 | 41.27 | .000 |
| bis24 | 1.84 | 0.02 | 86.05 | .000 | 1.72 | 0.04 | 45.10 | .000 | 1.66 | 0.02 | 67.88 | .000 | 1.78 | 0.04 | 45.98 | .000 | 1.77 | 0.04 | 45.04 | .000 | 1.97 | 0.06 | 30.85 | .000 |
| bis26 | 2.33 | 0.02 | 95.51 | .000 | 2.05 | 0.04 | 48.82 | .000 | 1.87 | 0.03 | 66.56 | .000 | 2.03 | 0.04 | 47.79 | .000 | 2.22 | 0.04 | 52.13 | .000 | 2.40 | 0.06 | 37.63 | .000 |
| bis28 | 1.98 | 0.02 | 80.10 | .000 | 1.82 | 0.04 | 42.55 | .000 | 1.80 | 0.03 | 56.96 | .000 | 1.93 | 0.05 | 41.28 | .000 | 1.84 | 0.04 | 41.76 | .000 | 1.95 | 0.07 | 29.32 | .000 |
| bis02 | 1.90 | 0.02 | 89.62 | .000 | 1.68 | 0.04 | 45.98 | .000 | 1.54 | 0.02 | 62.88 | .000 | 1.56 | 0.04 | 40.79 | .000 | 1.92 | 0.04 | 46.98 | .000 | 2.11 | 0.06 | 35.22 | .000 |
| bis03 | 2.57 | 0.02 | 104.89 | .000 | 2.23 | 0.04 | 54.62 | .000 | 2.70 | 0.03 | 93.41 | .000 | 2.54 | 0.04 | 58.41 | .000 | 2.21 | 0.04 | 50.83 | .000 | 2.35 | 0.06 | 36.29 | .000 |
| bis04 | 2.46 | 0.03 | 93.71 | .000 | 2.28 | 0.04 | 52.68 | .000 | 2.71 | 0.03 | 79.14 | .000 | 2.47 | 0.05 | 50.37 | .000 | 2.15 | 0.05 | 46.90 | .000 | 2.38 | 0.07 | 34.27 | .000 |
| bis16 | 1.70 | 0.02 | 79.53 | .000 | 1.76 | 0.04 | 41.69 | .000 | 1.47 | 0.02 | 63.80 | .000 | 1.65 | 0.04 | 41.95 | .000 | 1.70 | 0.04 | 41.99 | .000 | 1.85 | 0.06 | 29.02 | .000 |
| bis17 | 1.93 | 0.02 | 92.99 | .000 | 1.75 | 0.04 | 46.92 | .000 | 1.60 | 0.02 | 67.03 | .000 | 1.65 | 0.04 | 45.11 | .000 | 1.93 | 0.04 | 51.95 | .000 | 2.09 | 0.06 | 34.72 | .000 |
| bis19 | 1.99 | 0.02 | 94.05 | .000 | 1.75 | 0.03 | 51.17 | .000 | 1.66 | 0.02 | 67.14 | .000 | 1.73 | 0.04 | 45.07 | .000 | 1.92 | 0.04 | 49.85 | .000 | 2.06 | 0.06 | 37.38 | .000 |
| bis21 | 1.60 | 0.02 | 79.96 | .000 | 1.54 | 0.04 | 42.81 | .000 | 1.39 | 0.02 | 65.99 | .000 | 1.54 | 0.04 | 40.31 | .000 | 1.45 | 0.04 | 40.90 | .000 | 1.97 | 0.07 | 29.06 | .000 |
| bis22 | 2.02 | 0.02 | 94.69 | .000 | 1.82 | 0.04 | 46.39 | .000 | 1.72 | 0.03 | 68.01 | .000 | 1.71 | 0.04 | 46.52 | .000 | 2.02 | 0.04 | 46.77 | .000 | 1.92 | 0.06 | 30.01 | .000 |
| bis23 | 1.91 | 0.02 | 85.36 | .000 | 1.59 | 0.03 | 46.21 | .000 | 1.72 | 0.03 | 59.37 | .000 | 1.95 | 0.04 | 43.45 | .000 | 1.91 | 0.04 | 42.55 | .000 | 2.11 | 0.07 | 31.25 | .000 |
| bis25 | 1.79 | 0.02 | 72.87 | .000 | 1.66 | 0.04 | 38.65 | .000 | 1.49 | 0.03 | 57.64 | .000 | 1.60 | 0.04 | 37.92 | .000 | 1.63 | 0.04 | 38.24 | .000 | 1.60 | 0.07 | 23.89 | .000 |
| bis30 | 2.20 | 0.02 | 94.34 | .000 | 2.34 | 0.04 | 57.56 | .000 | 2.35 | 0.03 | 75.08 | .000 | 2.29 | 0.04 | 55.28 | .000 | 2.32 | 0.04 | 52.16 | .000 | 2.13 | 0.06 | 33.41 | .000 |
| bis01 | 2.03 | 0.02 | 90.65 | .000 | 2.07 | 0.04 | 53.45 | .000 | 1.94 | 0.03 | 71.58 | .000 | 1.77 | 0.04 | 46.43 | .000 | 2.10 | 0.04 | 48.47 | .000 | 2.06 | 0.06 | 33.41 | .000 |
| bis07 | 1.99 | 0.03 | 76.76 | .000 | 2.05 | 0.04 | 47.73 | .000 | 1.91 | 0.03 | 62.84 | .000 | 1.77 | 0.04 | 41.04 | .000 | 2.01 | 0.05 | 41.98 | .000 | 2.02 | 0.07 | 30.31 | .000 |
| bis08 | 1.92 | 0.02 | 88.35 | .000 | 2.10 | 0.04 | 55.58 | .000 | 2.00 | 0.03 | 74.30 | .000 | 1.77 | 0.04 | 46.45 | .000 | 2.13 | 0.04 | 50.47 | .000 | 2.10 | 0.06 | 34.33 | .000 |
| bis10 | 2.47 | 0.03 | 87.76 | .000 | 2.48 | 0.05 | 51.93 | .000 | 2.38 | 0.03 | 68.11 | .000 | 2.17 | 0.05 | 44.65 | .000 | 2.29 | 0.05 | 45.64 | .000 | 2.09 | 0.07 | 27.84 | .000 |
| bis12 | 1.80 | 0.02 | 88.84 | .000 | 1.91 | 0.03 | 55.45 | .000 | 1.88 | 0.03 | 72.02 | .000 | 1.69 | 0.04 | 47.57 | .000 | 1.98 | 0.04 | 50.62 | .000 | 1.98 | 0.05 | 36.60 | .000 |
| bis13 | 2.29 | 0.03 | 81.61 | .000 | 2.40 | 0.05 | 51.69 | .000 | 2.32 | 0.03 | 68.88 | .000 | 2.14 | 0.05 | 43.88 | .000 | 2.31 | 0.05 | 46.42 | .000 | 2.20 | 0.07 | 31.81 | .000 |
| bis14 | 1.92 | 0.02 | 86.57 | .000 | 1.72 | 0.04 | 44.81 | .000 | 1.59 | 0.03 | 61.39 | .000 | 1.68 | 0.04 | 42.72 | .000 | 1.95 | 0.04 | 49.68 | .000 | 1.98 | 0.06 | 34.47 | .000 |
| bis15 | 2.04 | 0.02 | 84.50 | .000 | 2.20 | 0.04 | 49.40 | .000 | 2.34 | 0.03 | 71.93 | .000 | 2.20 | 0.05 | 46.47 | .000 | 2.18 | 0.05 | 47.54 | .000 | 1.95 | 0.06 | 33.23 | .000 |
| bis18 | 1.86 | 0.02 | 83.83 | .000 | 1.72 | 0.04 | 43.10 | .000 | 1.74 | 0.03 | 62.97 | .000 | 1.76 | 0.04 | 42.74 | .000 | 1.80 | 0.04 | 45.40 | .000 | 1.72 | 0.06 | 30.71 | .000 |
| bis27 | 2.36 | 0.02 | 106.76 | .000 | 2.00 | 0.04 | 49.98 | .000 | 2.20 | 0.03 | 75.27 | .000 | 2.43 | 0.04 | 61.24 | .000 | 2.15 | 0.04 | 52.93 | .000 | 2.17 | 0.06 | 34.18 | .000 |
| bis29 | 1.95 | 0.02 | 78.25 | .000 | 2.09 | 0.04 | 47.49 | .000 | 2.18 | 0.03 | 69.26 | .000 | 2.01 | 0.05 | 44.24 | .000 | 2.12 | 0.05 | 46.01 | .000 | 2.02 | 0.07 | 30.76 | .000 |
|  | Residual Variances | | | | | | | | |
| bis05 | 0.41 | 0.02 | 22.88 | .000 | 0.30 | 0.02 | 14.13 | .000 | 0.20 | 0.01 | 18.58 | .000 | 0.34 | 0.03 | 13.28 | .000 | 0.37 | 0.03 | 12.73 | .000 | 0.30 | 0.04 | 7.46 | .000 |
| bis06 | 0.67 | 0.03 | 24.01 | .000 | 0.55 | 0.04 | 13.84 | .000 | 0.52 | 0.03 | 19.43 | .000 | 0.61 | 0.04 | 13.88 | .000 | 0.76 | 0.06 | 13.69 | .000 | 0.57 | 0.07 | 8.71 | .000 |
| bis09 | 0.47 | 0.02 | 22.16 | .000 | 0.45 | 0.03 | 12.80 | .000 | 0.33 | 0.02 | 16.29 | .000 | 0.43 | 0.03 | 12.85 | .000 | 0.37 | 0.04 | 10.49 | .000 | 0.42 | 0.05 | 8.52 | .000 |
| bis11 | 0.67 | 0.03 | 24.07 | .000 | 0.63 | 0.04 | 14.53 | .000 | 0.57 | 0.03 | 19.26 | .000 | 0.67 | 0.05 | 13.82 | .000 | 0.73 | 0.05 | 13.97 | .000 | 0.58 | 0.07 | 8.68 | .000 |
| bis20 | 0.45 | 0.02 | 24.19 | .000 | 0.41 | 0.03 | 13.93 | .000 | 0.27 | 0.02 | 17.11 | .000 | 0.37 | 0.03 | 13.21 | .000 | 0.30 | 0.03 | 11.19 | .000 | 0.33 | 0.04 | 8.47 | .000 |
| bis24 | 0.58 | 0.02 | 25.84 | .000 | 0.64 | 0.04 | 15.36 | .000 | 0.41 | 0.02 | 19.78 | .000 | 0.51 | 0.04 | 14.15 | .000 | 0.59 | 0.04 | 13.92 | .000 | 0.66 | 0.07 | 9.19 | .000 |
| bis26 | 0.64 | 0.03 | 24.56 | .000 | 0.58 | 0.04 | 14.28 | .000 | 0.46 | 0.02 | 19.14 | .000 | 0.51 | 0.04 | 13.60 | .000 | 0.67 | 0.05 | 13.82 | .000 | 0.55 | 0.06 | 8.74 | .000 |
| bis28 | 0.60 | 0.03 | 23.81 | .000 | 0.66 | 0.05 | 14.64 | .000 | 0.58 | 0.03 | 19.08 | .000 | 0.57 | 0.04 | 13.27 | .000 | 0.73 | 0.05 | 13.88 | .000 | 0.47 | 0.06 | 7.93 | .000 |
| bis02 | 0.35 | 0.02 | 22.43 | .000 | 0.39 | 0.03 | 14.00 | .000 | 0.21 | 0.01 | 16.67 | .000 | 0.33 | 0.03 | 12.85 | .000 | 0.46 | 0.04 | 12.91 | .000 | 0.45 | 0.05 | 8.71 | .000 |
| bis03 | 0.85 | 0.03 | 26.57 | .000 | 0.83 | 0.05 | 15.76 | .000 | 0.70 | 0.03 | 20.46 | .000 | 0.82 | 0.06 | 14.76 | .000 | 0.71 | 0.05 | 13.91 | .000 | 0.70 | 0.08 | 9.29 | .000 |
| bis04 | 0.98 | 0.04 | 26.58 | .000 | 0.93 | 0.06 | 15.76 | .000 | 0.98 | 0.05 | 20.47 | .000 | 1.05 | 0.07 | 14.78 | .000 | 0.82 | 0.06 | 13.99 | .000 | 0.74 | 0.08 | 9.13 | .000 |
| bis16 | 0.57 | 0.02 | 25.93 | .000 | 0.66 | 0.04 | 14.94 | .000 | 0.38 | 0.02 | 20.01 | .000 | 0.51 | 0.04 | 14.12 | .000 | 0.59 | 0.04 | 13.81 | .000 | 0.64 | 0.07 | 9.19 | .000 |
| bis17 | 0.25 | 0.01 | 19.38 | .000 | 0.23 | 0.02 | 10.84 | .000 | 0.14 | 0.01 | 14.07 | .000 | 0.24 | 0.02 | 11.70 | .000 | 0.15 | 0.02 | 7.03 | .000 | 0.22 | 0.04 | 6.21 | .000 |
| bis19 | 0.32 | 0.01 | 21.63 | .000 | 0.23 | 0.02 | 12.02 | .000 | 0.22 | 0.01 | 16.87 | .000 | 0.33 | 0.03 | 12.78 | .000 | 0.25 | 0.02 | 10.06 | .000 | 0.24 | 0.03 | 7.25 | .000 |
| bis21 | 0.53 | 0.02 | 26.23 | .000 | 0.53 | 0.03 | 15.29 | .000 | 0.34 | 0.02 | 20.20 | .000 | 0.43 | 0.03 | 13.72 | .000 | 0.46 | 0.03 | 13.82 | .000 | 0.80 | 0.09 | 9.36 | .000 |
| bis22 | 0.41 | 0.02 | 23.81 | .000 | 0.51 | 0.04 | 14.56 | .000 | 0.30 | 0.02 | 18.37 | .000 | 0.35 | 0.03 | 13.28 | .000 | 0.49 | 0.04 | 12.74 | .000 | 0.54 | 0.06 | 8.84 | .000 |
| bis23 | 0.71 | 0.03 | 26.55 | .000 | 0.57 | 0.04 | 15.69 | .000 | 0.65 | 0.03 | 20.25 | .000 | 0.75 | 0.05 | 14.42 | .000 | 0.79 | 0.06 | 14.04 | .000 | 0.80 | 0.09 | 9.38 | .000 |
| bis25 | 0.63 | 0.03 | 24.82 | .000 | 0.67 | 0.05 | 14.87 | .000 | 0.39 | 0.02 | 19.36 | .000 | 0.42 | 0.03 | 13.03 | .000 | 0.63 | 0.05 | 13.66 | .000 | 0.64 | 0.07 | 9.01 | .000 |
| bis30 | 0.71 | 0.03 | 26.16 | .000 | 0.76 | 0.05 | 15.57 | .000 | 0.74 | 0.04 | 20.19 | .000 | 0.74 | 0.05 | 14.74 | .000 | 0.78 | 0.06 | 14.02 | .000 | 0.65 | 0.07 | 9.22 | .000 |
| bis01 | 0.43 | 0.02 | 23.00 | .000 | 0.34 | 0.03 | 12.73 | .000 | 0.27 | 0.02 | 17.13 | .000 | 0.39 | 0.03 | 13.32 | .000 | 0.40 | 0.03 | 11.93 | .000 | 0.37 | 0.05 | 7.92 | .000 |
| bis07 | 0.72 | 0.03 | 24.73 | .000 | 0.60 | 0.04 | 14.42 | .000 | 0.45 | 0.02 | 18.71 | .000 | 0.52 | 0.04 | 13.52 | .000 | 0.62 | 0.05 | 12.90 | .000 | 0.53 | 0.06 | 8.55 | .000 |
| bis08 | 0.44 | 0.02 | 23.77 | .000 | 0.37 | 0.03 | 13.39 | .000 | 0.30 | 0.02 | 17.90 | .000 | 0.32 | 0.03 | 12.55 | .000 | 0.38 | 0.03 | 11.94 | .000 | 0.38 | 0.05 | 8.12 | .000 |
| bis10 | 0.85 | 0.03 | 24.80 | .000 | 0.86 | 0.06 | 14.99 | .000 | 0.74 | 0.04 | 19.53 | .000 | 0.79 | 0.06 | 14.10 | .000 | 0.76 | 0.06 | 13.25 | .000 | 0.80 | 0.09 | 8.97 | .000 |
| bis12 | 0.36 | 0.02 | 23.07 | .000 | 0.32 | 0.02 | 13.69 | .000 | 0.24 | 0.01 | 16.98 | .000 | 0.25 | 0.02 | 12.10 | .000 | 0.31 | 0.03 | 11.72 | .000 | 0.28 | 0.04 | 7.86 | .000 |
| bis13 | 0.80 | 0.03 | 24.45 | .000 | 0.57 | 0.04 | 13.53 | .000 | 0.57 | 0.03 | 18.77 | .000 | 0.74 | 0.05 | 13.86 | .000 | 0.66 | 0.05 | 12.81 | .000 | 0.61 | 0.07 | 8.71 | .000 |
| bis14 | 0.50 | 0.02 | 24.49 | .000 | 0.58 | 0.04 | 15.08 | .000 | 0.44 | 0.02 | 19.75 | .000 | 0.49 | 0.03 | 13.90 | .000 | 0.56 | 0.04 | 13.82 | .000 | 0.41 | 0.05 | 8.64 | .000 |
| bis15 | 0.77 | 0.03 | 26.23 | .000 | 0.92 | 0.06 | 15.60 | .000 | 0.77 | 0.04 | 20.09 | .000 | 0.85 | 0.06 | 14.45 | .000 | 0.70 | 0.05 | 13.58 | .000 | 0.57 | 0.06 | 9.29 | .000 |
| bis18 | 0.60 | 0.02 | 25.69 | .000 | 0.67 | 0.04 | 15.30 | .000 | 0.47 | 0.02 | 19.60 | .000 | 0.53 | 0.04 | 13.88 | .000 | 0.55 | 0.04 | 13.71 | .000 | 0.54 | 0.06 | 9.34 | .000 |
| bis27 | 0.67 | 0.03 | 26.41 | .000 | 0.76 | 0.05 | 15.64 | .000 | 0.66 | 0.03 | 20.25 | .000 | 0.65 | 0.04 | 14.63 | .000 | 0.64 | 0.05 | 14.02 | .000 | 0.67 | 0.07 | 9.27 | .000 |
| bis29 | 0.83 | 0.03 | 26.25 | .000 | 0.94 | 0.06 | 15.70 | .000 | 0.77 | 0.04 | 20.28 | .000 | 0.81 | 0.06 | 14.54 | .000 | 0.78 | 0.06 | 13.85 | .000 | 0.75 | 0.08 | 9.37 | .000 |
|  | Latent Intercepts | | | | | | | | |
| attentional | 0.00+ |  |  |  | 0.00+ |  |  |  | 0.00+ |  |  |  | 0.00+ |  |  |  | 0.00+ |  |  |  | 0.00+ |  |  |  |
| motor | 0.00+ |  |  |  | 0.00+ |  |  |  | 0.00+ |  |  |  | 0.00+ |  |  |  | 0.00+ |  |  |  | 0.00+ |  |  |  |
| nonplanning | 0.00+ |  |  |  | 0.00+ |  |  |  | 0.00+ |  |  |  | 0.00+ |  |  |  | 0.00+ |  |  |  | 0.00+ |  |  |  |
|  | Latent Variances | | | | | | | | |
| attentional | 1.00+ |  |  |  | 1.00+ |  |  |  | 1.00+ |  |  |  | 1.00+ |  |  |  | 1.00+ |  |  |  | 1.00+ |  |  |  |
| motor | 1.00+ |  |  |  | 1.00+ |  |  |  | 1.00+ |  |  |  | 1.00+ |  |  |  | 1.00+ |  |  |  | 1.00+ |  |  |  |
| nonplanning | 1.00+ |  |  |  | 1.00+ |  |  |  | 1.00+ |  |  |  | 1.00+ |  |  |  | 1.00+ |  |  |  | 1.00+ |  |  |  |
|  | Latent Covariances | | | | | | | | |
| attentional w/motor | 0.65 | 0.02 | 28.43 | .000 | 0.67 | 0.04 | 19.00 | .000 | 0.70 | 0.02 | 28.67 | .000 | 0.78 | 0.03 | 27.08 | .000 | 0.47 | 0.05 | 8.58 | .000 | 0.70 | 0.06 | 11.70 | .000 |
| attentional w/nonplanning | 0.73 | 0.02 | 34.28 | .000 | 0.72 | 0.03 | 22.38 | .000 | 0.83 | 0.02 | 45.63 | .000 | 0.81 | 0.03 | 29.63 | .000 | 0.88 | 0.03 | 27.57 | .000 | 0.72 | 0.06 | 12.11 | .000 |
| motor w/nonplanning | 0.74 | 0.02 | 38.28 | .000 | 0.65 | 0.03 | 18.88 | .000 | 0.71 | 0.02 | 31.35 | .000 | 0.69 | 0.03 | 20.10 | .000 | 0.43 | 0.05 | 8.16 | .000 | 0.77 | 0.05 | 15.12 | .000 |
|  | Fit Indices | | | | | | | | |
| χ2 | 17433.48(2412) |  |  | .000 |  |  |  |  |  |  |  |  |  |  |  |  |  |  |  |  |  |  |  |  |
| DF | 2412.00 |  |  |  |  |  |  |  |  |  |  |  |  |  |  |  |  |  |  |  |  |  |  |  |
| RMSEA | 0.10 |  |  |  |  |  |  |  |  |  |  |  |  |  |  |  |  |  |  |  |  |  |  |  |
| CFI | 0.60 |  |  |  |  |  |  |  |  |  |  |  |  |  |  |  |  |  |  |  |  |  |  |  |
| NNFI | 0.57 |  |  |  |  |  |  |  |  |  |  |  |  |  |  |  |  |  |  |  |  |  |  |  |
| SRMR | 0.10 |  |  |  |  |  |  |  |  |  |  |  |  |  |  |  |  |  |  |  |  |  |  |  |
| \_BOML10\_+Fixed parameter |

  
