# Supplementary material for: The relationship between apathy and impulsivity in large population samples
Source: Sci Rep. 2021 Mar 1;11:4830. doi: 10.1038/s41598-021-84364-w (PMC7921138; doi:10.1038/s41598-021-84364-w)
Supplement: Supplementary file 6 — Supplementary Information. [file 41598_2021_84364_MOESM6_ESM.html]

|  |  |  |  |  |
| --- | --- | --- | --- | --- |
|  | Model | | | |
|  | Estimate | Std. Err. | z | p |
|  | Factor Loadings | | | |
| negUrgency |
| uppsp02 | 0.59 | 0.03 | 21.85 | .000 |
| uppsp07 | 0.47 | 0.03 | 14.56 | .000 |
| uppsp12 | 0.56 | 0.03 | 20.80 | .000 |
| uppsp17 | 0.69 | 0.03 | 26.25 | .000 |
| uppsp22 | 0.68 | 0.03 | 24.47 | .000 |
| uppsp29 | 0.70 | 0.02 | 29.15 | .000 |
| uppsp34 | 0.61 | 0.03 | 21.03 | .000 |
| uppsp39 | 0.65 | 0.02 | 26.12 | .000 |
| uppsp44 | 0.72 | 0.03 | 28.38 | .000 |
| uppsp50 | 0.63 | 0.02 | 25.57 | .000 |
| uppsp53 | 0.65 | 0.02 | 28.03 | .000 |
| uppsp58 | 0.67 | 0.03 | 24.12 | .000 |
| posUrgency |
| uppsp05 | 0.58 | 0.02 | 24.52 | .000 |
| uppsp10 | 0.67 | 0.02 | 30.10 | .000 |
| uppsp15 | 0.67 | 0.02 | 31.08 | .000 |
| uppsp20 | 0.59 | 0.02 | 29.77 | .000 |
| uppsp25 | 0.61 | 0.02 | 29.32 | .000 |
| uppsp30 | 0.59 | 0.02 | 30.48 | .000 |
| uppsp35 | 0.53 | 0.02 | 27.43 | .000 |
| uppsp40 | 0.67 | 0.02 | 33.99 | .000 |
| uppsp45 | 0.64 | 0.02 | 31.28 | .000 |
| uppsp49 | 0.29 | 0.02 | 12.70 | .000 |
| uppsp52 | 0.09 | 0.04 | 2.23 | .026 |
| uppsp54 | 0.35 | 0.03 | 12.53 | .000 |
| uppsp57 | 0.43 | 0.03 | 14.68 | .000 |
| uppsp59 | 0.62 | 0.02 | 25.67 | .000 |
| lackPremeditation |
| uppsp01 | 0.34 | 0.03 | 11.89 | .000 |
| uppsp06 | 0.48 | 0.02 | 23.45 | .000 |
| uppsp11 | 0.40 | 0.03 | 11.49 | .000 |
| uppsp16 | 0.50 | 0.02 | 24.12 | .000 |
| uppsp21 | 0.35 | 0.03 | 13.43 | .000 |
| uppsp28 | 0.47 | 0.02 | 21.82 | .000 |
| uppsp33 | 0.50 | 0.02 | 24.26 | .000 |
| uppsp38 | 0.46 | 0.02 | 20.15 | .000 |
| uppsp43 | 0.42 | 0.02 | 18.46 | .000 |
| uppsp48 | 0.59 | 0.02 | 27.71 | .000 |
| uppsp55 | 0.37 | 0.03 | 14.42 | .000 |
| lackPerseverance |
| uppsp04 | 0.52 | 0.02 | 22.24 | .000 |
| uppsp09 | 0.61 | 0.03 | 22.58 | .000 |
| uppsp14 | 0.38 | 0.03 | 13.92 | .000 |
| uppsp19 | 0.32 | 0.02 | 12.86 | .000 |
| uppsp24 | 0.53 | 0.03 | 18.47 | .000 |
| uppsp27 | 0.67 | 0.02 | 30.98 | .000 |
| uppsp32 | 0.54 | 0.02 | 23.92 | .000 |
| uppsp37 | 0.63 | 0.02 | 29.05 | .000 |
| uppsp42 | 0.71 | 0.02 | 31.47 | .000 |
| uppsp47 | 0.59 | 0.03 | 18.58 | .000 |
| sensationSeeking |
| uppsp03 | 0.57 | 0.03 | 19.27 | .000 |
| uppsp08 | 0.60 | 0.03 | 20.23 | .000 |
| uppsp13 | 0.42 | 0.03 | 12.67 | .000 |
| uppsp18 | 0.68 | 0.04 | 19.23 | .000 |
| uppsp23 | 0.69 | 0.03 | 26.17 | .000 |
| uppsp26 | 0.74 | 0.03 | 21.44 | .000 |
| uppsp31 | 0.70 | 0.03 | 25.82 | .000 |
| uppsp36 | 0.72 | 0.04 | 19.42 | .000 |
| uppsp41 | 0.77 | 0.03 | 27.53 | .000 |
| uppsp46 | 0.80 | 0.03 | 23.45 | .000 |
| uppsp51 | 0.08 | 0.04 | 2.21 | .027 |
| uppsp56 | 0.72 | 0.04 | 19.59 | .000 |
|  | Intercepts | | | |
| uppsp02 | 1.81 | 0.03 | 60.03 | .000 |
| uppsp07 | 2.30 | 0.03 | 68.61 | .000 |
| uppsp12 | 2.08 | 0.03 | 70.01 | .000 |
| uppsp17 | 1.91 | 0.03 | 61.45 | .000 |
| uppsp22 | 1.92 | 0.03 | 59.79 | .000 |
| uppsp29 | 1.82 | 0.03 | 62.31 | .000 |
| uppsp34 | 1.98 | 0.03 | 61.14 | .000 |
| uppsp39 | 1.90 | 0.03 | 64.78 | .000 |
| uppsp44 | 1.76 | 0.03 | 57.57 | .000 |
| uppsp50 | 1.60 | 0.03 | 55.13 | .000 |
| uppsp53 | 1.65 | 0.03 | 58.91 | .000 |
| uppsp58 | 2.11 | 0.03 | 66.06 | .000 |
| uppsp05 | 1.62 | 0.03 | 58.86 | .000 |
| uppsp10 | 1.63 | 0.03 | 59.16 | .000 |
| uppsp15 | 1.56 | 0.03 | 57.65 | .000 |
| uppsp20 | 1.51 | 0.02 | 61.95 | .000 |
| uppsp25 | 1.53 | 0.03 | 60.29 | .000 |
| uppsp30 | 1.45 | 0.02 | 59.77 | .000 |
| uppsp35 | 1.42 | 0.02 | 61.09 | .000 |
| uppsp40 | 1.50 | 0.03 | 58.29 | .000 |
| uppsp45 | 1.51 | 0.03 | 58.34 | .000 |
| uppsp49 | 1.61 | 0.02 | 67.87 | .000 |
| uppsp52 | 2.62 | 0.04 | 65.68 | .000 |
| uppsp54 | 2.11 | 0.03 | 73.84 | .000 |
| uppsp57 | 2.30 | 0.03 | 74.15 | .000 |
| uppsp59 | 1.67 | 0.03 | 58.89 | .000 |
| uppsp01 | 2.00 | 0.03 | 69.78 | .000 |
| uppsp06 | 1.60 | 0.02 | 69.76 | .000 |
| uppsp11 | 1.88 | 0.03 | 54.37 | .000 |
| uppsp16 | 1.55 | 0.02 | 66.03 | .000 |
| uppsp21 | 1.98 | 0.03 | 74.45 | .000 |
| uppsp28 | 1.62 | 0.02 | 68.42 | .000 |
| uppsp33 | 1.59 | 0.02 | 67.96 | .000 |
| uppsp38 | 1.73 | 0.02 | 69.38 | .000 |
| uppsp43 | 1.64 | 0.02 | 68.16 | .000 |
| uppsp48 | 1.66 | 0.03 | 65.68 | .000 |
| uppsp55 | 1.57 | 0.03 | 59.89 | .000 |
| uppsp04 | 1.61 | 0.03 | 61.81 | .000 |
| uppsp09 | 1.90 | 0.03 | 62.76 | .000 |
| uppsp14 | 1.92 | 0.03 | 68.74 | .000 |
| uppsp19 | 1.85 | 0.03 | 73.37 | .000 |
| uppsp24 | 1.97 | 0.03 | 63.41 | .000 |
| uppsp27 | 1.70 | 0.03 | 63.67 | .000 |
| uppsp32 | 1.73 | 0.03 | 67.20 | .000 |
| uppsp37 | 1.72 | 0.03 | 65.16 | .000 |
| uppsp42 | 1.75 | 0.03 | 61.88 | .000 |
| uppsp47 | 2.04 | 0.03 | 60.05 | .000 |
| uppsp03 | 2.46 | 0.03 | 77.55 | .000 |
| uppsp08 | 2.44 | 0.03 | 75.99 | .000 |
| uppsp13 | 2.38 | 0.03 | 71.41 | .000 |
| uppsp18 | 2.43 | 0.04 | 63.82 | .000 |
| uppsp23 | 2.00 | 0.03 | 65.14 | .000 |
| uppsp26 | 1.93 | 0.04 | 50.84 | .000 |
| uppsp31 | 2.52 | 0.03 | 80.29 | .000 |
| uppsp36 | 2.23 | 0.04 | 56.04 | .000 |
| uppsp41 | 2.18 | 0.03 | 65.45 | .000 |
| uppsp46 | 2.19 | 0.04 | 56.97 | .000 |
| uppsp51 | 2.26 | 0.03 | 66.38 | .000 |
| uppsp56 | 2.42 | 0.04 | 60.95 | .000 |
|  | Residual Variances | | | |
| uppsp02 | 0.39 | 0.02 | 19.21 | .000 |
| uppsp07 | 0.69 | 0.03 | 19.80 | .000 |
| uppsp12 | 0.40 | 0.02 | 19.33 | .000 |
| uppsp17 | 0.31 | 0.02 | 18.50 | .000 |
| uppsp22 | 0.37 | 0.02 | 18.84 | .000 |
| uppsp29 | 0.21 | 0.01 | 17.68 | .000 |
| uppsp34 | 0.48 | 0.02 | 19.30 | .000 |
| uppsp39 | 0.27 | 0.01 | 18.53 | .000 |
| uppsp44 | 0.24 | 0.01 | 17.94 | .000 |
| uppsp50 | 0.28 | 0.01 | 18.64 | .000 |
| uppsp53 | 0.21 | 0.01 | 18.05 | .000 |
| uppsp58 | 0.38 | 0.02 | 18.90 | .000 |
| uppsp05 | 0.28 | 0.01 | 19.29 | .000 |
| uppsp10 | 0.17 | 0.01 | 18.32 | .000 |
| uppsp15 | 0.15 | 0.01 | 18.02 | .000 |
| uppsp20 | 0.14 | 0.01 | 18.41 | .000 |
| uppsp25 | 0.16 | 0.01 | 18.52 | .000 |
| uppsp30 | 0.13 | 0.01 | 18.21 | .000 |
| uppsp35 | 0.16 | 0.01 | 18.89 | .000 |
| uppsp40 | 0.09 | 0.01 | 16.56 | .000 |
| uppsp45 | 0.13 | 0.01 | 17.95 | .000 |
| uppsp49 | 0.37 | 0.02 | 19.97 | .000 |
| uppsp52 | 1.28 | 0.06 | 20.12 | .000 |
| uppsp54 | 0.54 | 0.03 | 19.97 | .000 |
| uppsp57 | 0.59 | 0.03 | 19.91 | .000 |
| uppsp59 | 0.27 | 0.01 | 19.15 | .000 |
| uppsp01 | 0.55 | 0.03 | 19.70 | .000 |
| uppsp06 | 0.20 | 0.01 | 17.78 | .000 |
| uppsp11 | 0.81 | 0.04 | 19.73 | .000 |
| uppsp16 | 0.20 | 0.01 | 17.56 | .000 |
| uppsp21 | 0.45 | 0.02 | 19.57 | .000 |
| uppsp28 | 0.23 | 0.01 | 18.24 | .000 |
| uppsp33 | 0.19 | 0.01 | 17.51 | .000 |
| uppsp38 | 0.29 | 0.02 | 18.62 | .000 |
| uppsp43 | 0.29 | 0.02 | 18.93 | .000 |
| uppsp48 | 0.17 | 0.01 | 15.92 | .000 |
| uppsp55 | 0.42 | 0.02 | 19.47 | .000 |
| uppsp04 | 0.28 | 0.02 | 18.81 | .000 |
| uppsp09 | 0.37 | 0.02 | 18.75 | .000 |
| uppsp14 | 0.49 | 0.02 | 19.72 | .000 |
| uppsp19 | 0.41 | 0.02 | 19.79 | .000 |
| uppsp24 | 0.50 | 0.03 | 19.33 | .000 |
| uppsp27 | 0.14 | 0.01 | 15.60 | .000 |
| uppsp32 | 0.25 | 0.01 | 18.48 | .000 |
| uppsp37 | 0.17 | 0.01 | 16.79 | .000 |
| uppsp42 | 0.14 | 0.01 | 15.21 | .000 |
| uppsp47 | 0.59 | 0.03 | 19.32 | .000 |
| uppsp03 | 0.49 | 0.03 | 18.94 | .000 |
| uppsp08 | 0.48 | 0.03 | 18.77 | .000 |
| uppsp13 | 0.73 | 0.04 | 19.69 | .000 |
| uppsp18 | 0.71 | 0.04 | 18.94 | .000 |
| uppsp23 | 0.29 | 0.02 | 17.14 | .000 |
| uppsp26 | 0.62 | 0.03 | 18.54 | .000 |
| uppsp31 | 0.31 | 0.02 | 17.28 | .000 |
| uppsp36 | 0.77 | 0.04 | 18.91 | .000 |
| uppsp41 | 0.30 | 0.02 | 16.51 | .000 |
| uppsp46 | 0.56 | 0.03 | 18.05 | .000 |
| uppsp51 | 0.94 | 0.05 | 20.11 | .000 |
| uppsp56 | 0.76 | 0.04 | 18.88 | .000 |
|  | Latent Intercepts | | | |
| negUrgency | 0.00+ |  |  |  |
| posUrgency | 0.00+ |  |  |  |
| lackPremeditation | 0.00+ |  |  |  |
| lackPerseverance | 0.00+ |  |  |  |
| sensationSeeking | 0.00+ |  |  |  |
|  | Latent Variances | | | |
| negUrgency | 1.00+ |  |  |  |
| posUrgency | 1.00+ |  |  |  |
| lackPremeditation | 1.00+ |  |  |  |
| lackPerseverance | 1.00+ |  |  |  |
| sensationSeeking | 1.00+ |  |  |  |
|  | Latent Covariances | | | |
| negUrgency w/posUrgency | 0.86 | 0.01 | 77.09 | .000 |
| negUrgency w/lackPremeditation | 0.61 | 0.03 | 24.27 | .000 |
| negUrgency w/lackPerseverance | 0.58 | 0.03 | 22.40 | .000 |
| negUrgency w/sensationSeeking | 0.28 | 0.04 | 7.87 | .000 |
| posUrgency w/lackPremeditation | 0.61 | 0.02 | 24.72 | .000 |
| posUrgency w/lackPerseverance | 0.43 | 0.03 | 14.00 | .000 |
| posUrgency w/sensationSeeking | 0.34 | 0.03 | 10.35 | .000 |
| lackPremeditation w/lackPerseverance | 0.50 | 0.03 | 16.94 | .000 |
| lackPremeditation w/sensationSeeking | 0.38 | 0.03 | 11.42 | .000 |
| lackPerseverance w/sensationSeeking | -0.03 | 0.04 | -0.73 | .467 |
|  | Fit Indices | | | |
| χ2 | 8155.19(1642) |  |  | .000 |
| DF | 1642.00 |  |  |  |
| RMSEA | 0.07 |  |  |  |
| CFI | 0.80 |  |  |  |
| NNFI | 0.79 |  |  |  |
| SRMR | 0.11 |  |  |  |
| +Fixed parameter | | | | |

  
