# Supplementary material for: The relationship between apathy and impulsivity in large population samples
Source: Sci Rep. 2021 Mar 1;11:4830. doi: 10.1038/s41598-021-84364-w (PMC7921138; doi:10.1038/s41598-021-84364-w)
Supplement: Supplementary file 7 — Supplementary Information. [file 41598_2021_84364_MOESM7_ESM.html]

|  |  |  |  |  |
| --- | --- | --- | --- | --- |
|  | Model | | | |
|  | Estimate | Std. Err. | z | p |
|  | Factor Loadings | | | |
| factor01 |
| BISmotor | 2.43 | 0.86 | 2.83 | .005 |
| BISattention | 2.66 | 0.29 | 9.22 | .000 |
| BISnp | 3.41 | 0.35 | 9.87 | .000 |
| AMIbeh | 0.53 | 0.12 | 4.31 | .000 |
| factor02 |
| AMIemo | 0.59 | 0.03 | 18.76 | .000 |
| factor03 |
| AMIbeh | 0.33 | 0.12 | 2.76 | .006 |
| AMIsoc | 0.22 | 0.06 | 3.40 | .001 |
| BISmotor | -3.16 | 0.87 | -3.64 | .000 |
|  | Intercepts | | | |
| BISmotor | 22.57 | 0.33 | 67.71 | .000 |
| BISattention | 17.01 | 0.30 | 57.58 | .000 |
| BISnp | 22.29 | 0.36 | 62.70 | .000 |
| AMIbeh | 1.46 | 0.06 | 26.09 | .000 |
| AMIemo | 1.05 | 0.04 | 23.42 | .000 |
| AMIsoc | 1.49 | 0.05 | 29.52 | .000 |
|  | Residual Variances | | | |
| BISmotor | -0.72 | 5.22 | -0.14 | .890 |
| BISattention | 8.29 | 1.11 | 7.47 | .000 |
| BISnp | 10.59 | 1.58 | 6.70 | .000 |
| AMIbeh | 0.26 | 0.08 | 3.39 | .001 |
| AMIemo | 0.00+ |  |  |  |
| AMIsoc | 0.40 | 0.05 | 8.66 | .000 |
|  | Latent Intercepts | | | |
| factor01 | 0.00+ |  |  |  |
| factor02 | 0.00+ |  |  |  |
| factor03 | 0.00+ |  |  |  |
|  | Latent Variances | | | |
| factor01 | 1.00+ |  |  |  |
| factor02 | 1.00+ |  |  |  |
| factor03 | 1.00+ |  |  |  |
|  | Latent Covariances | | | |
| factor01 w/factor02 | 0.05 | 0.08 | 0.55 | .583 |
| factor01 w/factor03 | -0.28 | 0.27 | -1.04 | .298 |
| factor02 w/factor03 | -0.05 | 0.08 | -0.68 | .499 |
|  | Fit Indices | | | |
| χ2 | 18.83(5) |  |  | .002 |
| DF | 5.00 |  |  |  |
| RMSEA | 0.13 |  |  |  |
| CFI | 0.93 |  |  |  |
| NNFI | 0.80 |  |  |  |
| SRMR | 0.05 |  |  |  |
| +Fixed parameter | | | | |

  
